# Chemical direct conversion of human fibroblasts to mesenchymal stem cells that can alleviate inflammation in vivo

**DOI:** 10.1186/s13287-025-04605-x

**Published:** 2025-10-30

**Authors:** Kenta Yamamoto, Tsunao Kishida, Toshiro Yamamoto, Yoshihiro Sowa, Makoto Seki, Osam Mazda

**Affiliations:** 1 Department of Immunology, Kyoto Prefectural University of Medicine, Kamigyo, Kyoto 602-8566 Japan; 2 Department of Dental Medicine, Kyoto Prefectural University of Medicine, Kamigyo, Kyoto 602-8566 Japan; 3 Department of Plastic Surgery, Jichi Medical University, Tochigi, 329-0431 Japan; 4 Cellaxia Inc. Nihonbashi, Chuou, Tokyo 103-0012 Japan

**Keywords:** Direct reprogramming, Mesenchymal stem cells, Regenerative medicine

## Abstract

**Background:**

Mesenchymal stem cell (MSC) transplantation may significantly benefit patients with some inflammatory diseases. However, invasive procedures are required to collect autologous MSCs from patients, while in vitro expansion of MSCs may spoil their stemness. In this study, we aimed to induce MSC-like phenotypes in human dermal fibroblasts (HDFs).

**Methods:**

HDFs were cultured with some chemical compounds. The resultant cells were examined for their gene expression and multi-differentiation abilities in vitro. Anti-inflammatory functions in vivo were tested using two types of disease models in mice. Exosomes derived from the cells were also characterized.

**Results:**

A combination of a TGF-β receptor inhibitor, a ROCK inhibitor, and an ATM inhibitor provoked HDFs to strongly express MSC markers. The chemical compound-driven directly converted MSCs (cdMSCs) had multilineage differentiation potentials to osteogenic, chondrogenic, and adipogenic lineages in vitro. The genes related to TGF-β, MAPK, Hedgehog and WNT signaling pathways were remarkably changed in expression levels, while CpG DNA methylation statuses were also altered, during the cell type transition. The cdMSCs ameliorated both acute and chronic inflammatory diseases after transplantation into murine models of LPS-induced lung injury and autoimmune arthritis. The cdMSCs secreted exosomes that promoted polarization of M0 macrophages to M2 phenotype while suppressing M1 macrophage induction in vitro.

**Conclusion:**

The three compounds successfully converted HDFs into MSC-like cells with high anti-inflammatory activities, which would be useful in regenerative medicine for inflammatory disease.

**Supplementary Information:**

The online version contains supplementary material available at 10.1186/s13287-025-04605-x.

## Background

Mesenchymal stem cells (MSCs) are known as multi-potent cells capable of differentiating into various mesenchymal linages including osteoblasts, adipocytes and chondrocytes [1, 2], while they could also trans-differentiate into the cells of other lineages such as neurons and cardiomyocytes under specific conditions [3, 4]. In addition to such multi-differentiation potential, MSCs exert anti-inflammatory and immunomodulatory functions through, at least partly, secretion of exosomes [5, 6], and produce cytokines and growth factors that promote tissue repair [7, 8]. Therefore, MSCs have been considered as promising cells for regenerative medicine and several previous reports have confirmed significant efficacies of MSC transplantation for several disease models such as bone injury [9], lung injury [10], rheumatoid arthritis [11], multiple sclerosis [12] and graft-versus-host disease (GVHD) [13]. In several clinical trials, autologous MSCs were transplanted to patients with GVHD, systemic lupus erythematosus (SLE), multiple sclerosis, diabetes, COVID19 and bone diseases [13–16].

However, cell therapy using MSCs has not been fully established as a standard therapy yet. One of the reasons is that autologous MSCs show different behavior depending on patient’s age and cell state when they are transplanted into the patients. Generally, donor age affects the MSC properties, and proliferation and differentiation capacities of MSCs decline with age of the donors [17, 18]. Because a limited number of MSCs can be practically obtained from a donor, they need to be expanded in vitro to gain an enough number of cells for transplantation, but MSCs undergo cellular senescence and lose their stem cell properties and tissue repair potentials during the expansion in vitro, which may lead to reduction in therapeutic efficacies of the MSCs [18, 19]. Also, as MSCs are usually collected from bone marrow or adipose tissue, the cell collection procedures are more or less invasive to patients. To overcome these drawbacks associated with autologous MSC collection, we propose a novel strategy in which MSCs are prepared by direct conversion (direct reprogramming) from another lineage of somatic cells such as fibroblasts that can be safely collected from any patients and easily expanded to a large number. The direct conversion technology has accomplished induction of various tissue cells from fibroblasts by transducing specific transcriptional factors [20–25]. We reported that functional osteoblasts, brown adipocytes, Schwann cells and urothelial cells were directly converted from human dermal fibroblasts (HDFs) by transducing them with specific sets of transcription factor genes [22–25]. The direct reprogramming may also be caused by culturing HDFs with some particular small molecular compounds, and such “chemical direct reprogramming” methods are advantageous over gene transfer-mediated methods in terms of the absence of potential risk of tumorigenicity associated with exogenous genes, ease of mass production and preservation of the chemical compounds, easy control of concentrations and reaction time, lack of immunogenicity, and low economic burden [26, 27]. Although the molecular mechanisms of cell fate conversion caused by compounds have not been well clarified, previous studies reported that epigenetic reprogramming is facilitated by some signaling pathway inhibitors, including histone deacetylase inhibitors, MAPK inhibitors, transforming growth factor-β receptor (TGF-β R) inhibitors, Rho-associated kinase (ROCK) inhibitors, bromodomain and extraterminal (BET) inhibitors, WNT inhibitors and GSK3 inhibitors [27–29]. We previously found that one of the TGF-β R inhibitors, activin receptor-like kinase 5 inhibitor II (ALK5iII) (Repsox), successfully promoted direct conversion of HDFs to osteoblasts [30].

In the present study, we succeeded in inducing MSC-like phenotypes in HDFs using chemical compounds, and analyzed characters and functions in vitro and in vivo of the resultant chemical compounds-driven directly converted MSCs (cdMSCs).

## Materials and Methods

### Cells

Normal human dermal fibroblasts derived from an abdominal dermal tissue of 45-years-old female (HDFs), breast dermal tissues of 18-years-old male (HDF18) and 22-years-old female (HDF22) were purchased from KURABO (Kurashiki, Japan) or Lifeline Cell Technology (San Diego, CA, USA). Fibroblasts were cultured in Dulbecco’s modified Eagle’s medium (DMEM) supplemented with 100 mM non-essential amino acids, 100 U/mL penicillin, 100 μg/mL streptomycin and 10% fetal bovine serum (FBS) [complete medium (CM)]. Normal human mesenchymal stem cells (MSCs) derived from adipose tissue of a female (ATMSCs), bone marrow of a 25-years-old male (BMMSCs), and a dental pulp tissue of a 29-years-old female (DPMSCs) were purchased from Lifeline Cell Technology, Cellular Engineering Technologies Inc. (Coralville, IA, USA) and Lonza (Basel, Switzerland), respectively. Culture medium for MSCs (Stem Cell Medium (SCM), StemLife MSC) was purchased from Lifeline Cell Technology (Frederick, MD, USA).

### Cell culture and induction of MSC characters

HDFs were cultured in the CM, which was replaced by fresh one every 3 to 4 days. MSCs were maintained in the SCM. To induce MSC characters in fibroblasts, HDFs were resuspended in the CM and seeded onto 24-well plates, 6-well plate plates, 60 mm culture dish or 100 mm culture dish (day-1). On the next day, the culture medium was replaced by the CM that contain 32 μM ALK5i (A) (Stem RD, #ALK-010), 1 μM Fasudil (F) (Selleck, #S1573) and/or 1 μM KU-60019 (K) (Sellek, #S1570) and cultured for 7 to 14 days. The culture medium was replaced by fresh one every 3 to 4 days.

### Flowcytometry

For cell surface marker analysis, cells were incubated with fluorescence-labeled antibodies shown in the Table S1. After incubation at 4℃ for 1 h, the cells were analyzed using FACS Canto III flow cytometer (BD, Flanklin Lakes, NJ, USA), and the acquired data were analyzed by FlowJo software (BD). For the experiments of Th17 and regulatory T cell detection, mouse splenocytes were Fc-blocked by mouse CD16/CD32 antibody (Biolegend, #101301) and incubated with Pacific blue-conjugated -mouse CD3 (Biolegend, #100213) and APC-conjugated-mouse CD4 (Biolegend, #100515) antibodies for 30 min at 4 °C. The cells were then pre-treated with Foxp3/Transcription Staining Buffer Set (eBioscience) to fix and permeabilize for intracellular staining, followed by incubation with PE-conjugated anti-mouse IL17A (Biolegend, #506903) or anti-mouse FoxP3 (Biolegend, #126403) antibodies for 60 min at 4°C. The cells were then analyzed using FACS Canto II flow cytometer (BD), and the acquired data were analyzed by FlowJo software (BD).

### RNA-seq analysis

Total RNA was extracted from cells using QIAcube and RNeasy (QIAGEN, Venlo, Netherland) with DNase (Thermo Fisher Science) treatment. Sequencing libraries were generated using the TruSeq Stranded mRNA Library Prep Kit (Illumina, San Diego, CA, USA) and applied to a NovaSeq 6000 sequencer (Illumina) for a pair-end 100 bp reads sequencing run. RNA-seq reads were aligned to the human reference genome (Rufseq), and acquired data were analyzed using Strand NGS software (Agilent Technologies, Santa Clara, CA, USA) and GSEA software (https://www.gsea-msigdb.org/gsea/index.jsp). RNA-Seq data have been deposited in GEO at NCBI under accession code GSE25005.

### Tri-lineage differentiation

Osteogenic medium (OGM) consisted of the CM supplemented with 50 μg/mL ascorbic acid, 10 mM β-glycerol phosphate, and 100 nM dexamethasone. For osteogenic differentiation, cells were seeded in 24-well plates and cultured in OGM for 14 to 21 days. Adipogenic (AGM) medium consisted of the CM supplemented with 10 μg/mL insulin, 1 μM dexamethasone and 1 mM IBMX. For adipogenic differentiation, cells were cultured in AGM in 24-well plates for 14 to 21 days. Chondrogenic medium (CGM) consisted of DMEM supplemented with 1% FBS, 1% ITS, 50 μg/mL ascorbic acid, and 10 ng/mL each of BMP-2, b-FGF, TGF-β and GDF5. For chondrogenic differentiation, cells were seeded at the center of the wells of 24-well plates. After incubation for 6 h, CGM was added to the wells and cells were cultured for 21 days by micro mass method.

### ALP activity assay

Cells were treated with 0.1% Triton X-100 (Nacalai Tesque) and ALP activity in the cell lysate was evaluated using LabAssay ALP ELISA kit (FUJIFILM, Tokyo, Japan). The ALP activity of each sample was normalized in accordance with the amount of protein that was assessed using BCA Protein Assay kit (TAKARA, Kusatsu, Japan).

### Cell staining

For Alizarin Red S staining, cells were fixed with 10% formaldehyde, followed by staining with Alizarin Red S solution (Sigma Aldrich). For quantitative measurement, the stain was solubilized into Triton-X and optical density (OD) of each solution was measured at 550 nm by Emax microplate reader. ALP staining and BODIPY fluorescent staining was performed using ALP staining kit (Sigma Aldrich) and BODIPY lipid probe (Thermo Fisher Science), respectively. For Oil Red O staining, cells were fixed with 10% formaldehyde, followed by staining with Oil Red O solution (Muto pure chemicals, Tokyo, Japan). For quantitative measurement, the cells were washed with 60% isopropanol, dissolved in 100% isopropanol, and OD of each solution was measured at 492 nm. For Alcian blue staining, cells were fixed with acetic acid solution, followed by staining with Alcian blue solution (SkyTek laboratories Inc., Logan, UT, USA). The cells were observed under a multipurpose fluoresces microscope (BZ-X710; Keyence, Osaka, Japan).

### Real time RT-PCR

Total RNA was extracted from cells using QIAcube and RNeasy (QIAGEN, Venlo, Netherland), and reverse-transcribed using ReverTra Ace qPCR RT Master Mix (Toyobo). The resultant cDNA was mixed with Real-Time PCR Master Mix (Applied Biosystems, Waltham, MA, USA) and matching probes/primers shown in Table S2. Real time PCR was carried out on a Step One Plus Real-Time PCR System (Applied Biosystems). All values (average ± SD) were normalized with respect to the human GAPDH or mouse β-actin mRNA level in each sample.

### DNA methylation analysis

The CpG DNA methylation statuses at the CD34 and CD140b gene upstream regions were analyzed as follows. The chromosomal DNA was isolated from HDFs, cdMSCs and ATMSCs using QIAcube and DNeasy (QIAGEN). After digestion by Mse I restriction enzyme (New England BioLabs, Ipswich, MA, U.S.A.), methylated and unmethylated DNA fragments were separated using MethylCollector Ultra Kit (Active Motif, Carlsbad, CA, U.S.A.) in accordance with manufacture’s instruction. Following purification by MinElute Reaction Cleanup Kit (QIAGEN), the samples were subjected to Real-Time PCR using probes/primers specific for the CD34 and CD140b gene upstream regions (Shown in Table S2). The relative copy numbers in methylated and unmethylated samples were calculated by 2-ΔCt method based on the corresponding ct values, and the relative methylation/unmethylation ratios were calculated (the ratio for HDFs was set to 1.0).

### Anti-inflammatory effects in vitro

A monocytic cell line, THP-1 (Invivogen, San Diego, CA, USA) was stimulated with 10 ng/mL LPS (Sigma) for 72 h in the presence/absence of HDFs, cdMSCs, or ATMSCs. Culture supernatants were collected and subjected to ELISA to measure concentrations of IL-1β using an ELISA kit (R&D Systems).

### Migration assay

Migration assay was conducted using migration assay kit (QCM 24-well Colorimetric Cell Migration Assay, Millipore, Burlington, MA, USA). Cells were resuspended in DMEM supplemented with 1% FBS and seeded on upper surface of transwell chamber membrane (5 × 104 cells/well), while DMEM supplemented with 20% FBS was added to the lower chambers. After culturing at 37 °C for 1 day, cells on the upper surface of the membrane were removed, and the cells that had migrated to the lower surface of membrane were fixed, stained, and observed under a multipurpose fluorescence microscope (BZ-X710). The stained chambers were then soaked in extraction buffer, and OD value at 550 nm of each extract was measured using a Microplate reader.

### LPS-induced acute lung injury (ALI) in mice and cell transplantation

All animal experiments were approved by the institutional Animal Experiment Committee, and all animal care was provided in accordance with institutional guidelines. NOG mice (Charles River) at 12 to 15 weeks of age were randomly divided into five Groups: (1) Control mice, (2) ALI mice administered with PBS, (3) ALI mice transplanted with HDFs, (4) ALI mice transplanted with cdMSCs, (5) ALI mice transplanted with ATMSCs. All mice were anesthetized with midazolam (4 mg/kg BW), medetomidine (0.75 mg/kg BW) and butorphanol (5 mg/kg BW) via intraperitoneal injections. Group 1 mice were intratracheally administered with 30 μL PBS instead of LPS. For the Groups 2 to 5 mice, ALI was induced by an intratracheal infusion of 30 μL LPS from *E*. *coli* O55:B5 (Sigma) at 10 mg/kg BW. Three hours later, mice were intratracheally infused with 30 μL PBS (Group 2), HDFs (5 × 10^5^ cells in 30 μL PBS) (Group 3), cdMSCs (5 × 10^5^ cells in 30 μL PBS) (Group 4), and ADMSCs (5 × 10^5^ cells in 30 μL PBS) (Group 5). Mice were then recovered from the anesthesia by injecting narcotic antagonist atipamezole (0.75 mg/kg BW). After 48 h the mice were sacrificed by barbituric over-dose, and BALF and lung tissue were collected. Cell number in BALF was counted by automated cell counter (Countess II, Thermo Fisher Science), and cytokine concentrations (mouse IL-6 and TNF-α) in the BALF were measured by ELISA kits (R & D Systems, Minneapolis, MN, USA). Lung tissue specimen was subjected to real time RT-PCR and histological assessment. For the histological assessment the specimen was fixed with 10% neutral-buffered formalin, embedded in paraffin, cut into 4-μm sections and stained with H & E. Degrees of alveolar congestion, neutrophil infiltration and alveolar wall thickening were scored as below. 0 = minimal injury, 1 = injury up to 25% of the field, 2 = injury up to 50% of the field, 3 = injury up to 75% of the field, and 4 = diffuse injury. Sum of the three categories’ scores is regarded as the lung injury score. The work has been reported in line with the ARRIVE guidelines 2.0.

### Rheumatoid arthritis in mice and cell transplantation

All animal experiments were approved by the institutional Animal Experiment Committee, and all animal care was provided in accordance with institutional guidelines. Female SKG/Jcl mice at 6- to 8-weeks old were purchased from CLEA Japan (Tokyo, Japan). They have a point mutation in ZAP-70 gene on the BALB/c background [31]. Mice were randomly divided into five groups: (1) Control mice, (2) arthritis mice administered with PBS, (3) arthritis mice transplanted with HDFs, (4) arthritis mice transplanted with cdMSCs, (5) arthritis mice transplanted with ADMSCs. Group 1 mice were SKG/jcl that did not manifest arthritis because they were administered with PBS instead of laminarin. Groups 2 to 5 SKG/jcl mice were intraperitoneally injected with laminarin (30 mg/mouse) (Nacalai tesque) twice to induce arthritis. One week after the second laminarin administration, the mice were intraperitoneally injected with 100 μL PBS (Group 2), HDFs (1 × 10^6^ cells in 100 μL PBS) (Group 3), cdMSCs (1 × 10^6^ cells in 100 μL PBS) (Group 4) and ADMSCs (1 × 10^6^ cells in 100 μL PBS) (Group 5) for five consecutive days as previously described [32]. Macroscopic assessment of arthritis was performed as follows: 0, no joint swelling; 0.1, swelling of one finger joint; 0.5, mild swelling of wrist or ankle; 1.0, severe swelling of wrist or ankle; swelling of one toe joint. Scores for all fingers of fore paws and hind paws, wrists and ankles were added for each mouse at 3 and 5 weeks after the laminarin administration. At 5 weeks after induction, the mice were sacrificed by barbituric over-dose, and the spleen, fore limbs and hind limbs were harvested. The fore limb specimen was used for gene expression analysis by real time RT-PCR, and hind limb tissues were subjected to micro CT examination (Scan Xmate-L090; Com Scan Techno) and histological assessment. For volumetric quantification of μCT images, damaged bone surface area was analyzed using ImageJ software. For histological assessment, hind limb tissues were fixed with 10% neutral-buffered formalin, and the samples were decalcified in 0.1 M EDTA at RT for 2 weeks, embedded in paraffin, cut into 4-μm sections and stained with H&E and Safranin O. Degree of tissue injury was scored as previously described [33]. In brief, degrees of inflammation were scored as follow; 0 = no inflammation, 1 = slight thickening of the tissue or some infiltrating cells into tissue, 2 = slight thickening of the tissue and some infiltrating cells into tissue, 3 = moderate thickening of the tissue and marked infiltrating cells into tissue, 4 = sever thickening of the tissue and highly infiltrating cells into tissue and exudation in to the joint cavity. Splenocytes were collected from the mice and cultured in RPMI medium supplemented with 100 mM non-essential amino acids, 100 U/mL penicillin, 100 μg/mL streptomycin, 10% FBS (RPMI-CM) and either Cell Stimulation Cocktail (plus a protein transporter inhibitors) (eBioscience, San Diego, CA, USA) or CD3/28 beads (VERITAS, Santa Clara, CA, USA). Contents of Th17 and regulatory T cells were analyzed by flowcytometry.

### In vivo imaging of fluorescence-labeled cdMSCs

Female SKG/Jcl mice at 6-weeks old were given twice intraperitoneal injections with laminarin (30 mg/mouse). One week after the second laminarin administration, the mice were intraperitoneally injected with cdMSCs (1 × 10^6^ cells) that had been labelled with 10 μM XenoLight DiR (Biotium, Fremont, CA, USA) for 20 min at 37 °C. After the anesthesia of the mice by isoflurane, the DiR-labeled cdMSCs were monitored using an IVIS Imaging System LUMINA III (PerkinElmer, Waltham, MA, USA) with 740 nm excitation and 790 nm emission at 1 h to 21 days after cell transplantation.

### Isolation and characterization of exosomes

Cells were washed with PBS for three times and cultured in culture medium in which FBS was replaced by exosome-depleted FBS (System Bioscience, Palo Alto, CA, USA.). Fourty-eight hours later, the culture supernatants were collected in low-protein adsorption tubes (PROTEOSAVE, Tokyo, Japan) and consecutively centrifuged three times at 300 × g for 5 min, 1200 × g for 20 min, and 10000 × g for 30 min to remove cells, debris and large extracellular vesicles. Exosomes were isolated from the supernatants by affinity reaction using a Mag Capture Exosome Isolation Kit PS Ver. 2 (FUJIFILM, Osaka, Japan). The sizes and concentrations of the isolated exosomes were measured by nanoparticle tracking analysis (NTA) (Nanosight NS300, Japan Quantum Design, Tokyo, Japan) as described elsewhere. The expression of exosomal markers CD9 and CD81 were examined by flowcytometry. In brief, exosomes were incubated with CD9 or CD81 magnetic beads (Dynabeads Human CD9 for Flow Detection or Dynabeads Human CD81 for Flow Detection, Thermo Fisher Science) for 16 h at 4 °C. The beads were washed, followed by incubation with PE-conjugated human CD9 or APC-conjugated human CD81 antibodies for 60 min at 4°C. Data were analyzed by FACS Canto II, and FlowJo software. The collected exosomes derived from HDFs, cdMSCs, and ADMSCs were referred to as HDF-exo, cdMSC-exo, and ADMSC-exo, respectively.

### Effects of exosomes on macrophage polarization

A monocytic cell line THP-1 (Invivogen, San Diego, CA, USA) was activated into M0 macrophages by stimulating in RPMI-CM supplemented with 100 ng/mL phorbol-12-myristate-13-acetate (PMA) (Calbiochem, Temecula, CA, USA) for 24 h, before induction into M1 and M2 phenotypes. For M1 macrophage induction, the cells were further cultured in RPMI-CM supplemented with 10 ng/mL LPS (Sigma) plus 20 ng/mL IFN-γ (PeproTech) for 48 h in the presence or absence of HDF-exo, cdMSC-exo, or ADMSC-exo. Culture supernatants were subjected to ELISA analysis to measure concentrations of IL-6 and TNF-α using an ELISA kit (R&D Systems). For M2 macrophage induction, the M0 macrophages were cultured in RPMI-CM supplemented with 20 ng/mL IL-4 (PeproTech) for 48 h with/without HDF-exo, cdMSC-exo, or ADMSC-exo. RNA was extracted from the cells, and mRNA levels for MRC-1 and IDO-1 genes were evaluated by real-time RT-PCR.

### Visualization of exosome uptake by M0 macrophages

Exosomes were labeled with a fluorescent dye using ExoSpalker Exosome membrane labeling kit (DOJINDO, Kumamoto, Japan). M0 macrophages induced as above were inculated with unlabeled or labeled exosomes, and 24 h later, cells were visualized under a fluorescent microscope.

### Statistical analysis

Data are expressed as mean ± standard deviation (S.D.). Data on replicates (n) is given in the each figure legends. Statistical significance was analyzed using Student’s t-test and ANOVA with Tukey–Kramer post hoc test. *P* < 0.05 was considered significant.

## Results

### MSC-like phenotypes were induced in HDFs that were treated with ALK5iII, Fasudil and KU-60019

We previously reported that osteoblast-like characters were induced in HDFs that were cultured in osteogenic medium (OGM) supplemented with ALK5iII (A) [30] (Supplementary Figures S1A–C). Through the experiments, we noticed that adipocyte-like characters were also induced in HDFs after they were cultured in adipogenic medium (AGM) supplemented with the same compound (Supplementary Figures S1D–E). Because both osteoblasts and adipocytes can be differentiated from MSCs, we thought of the possibility that ALK5ill can confer MSC-like phenotypes in HDFs. Moreover, we have found that an addition of either Fasudil (F) or KU-60019 (K) followed by culture in OGM and AGM also induced osteoblastic and adipogenic phenotypes in HDFs, respectively (Supplementary Figures S1F–G).

**Fig. 1:**
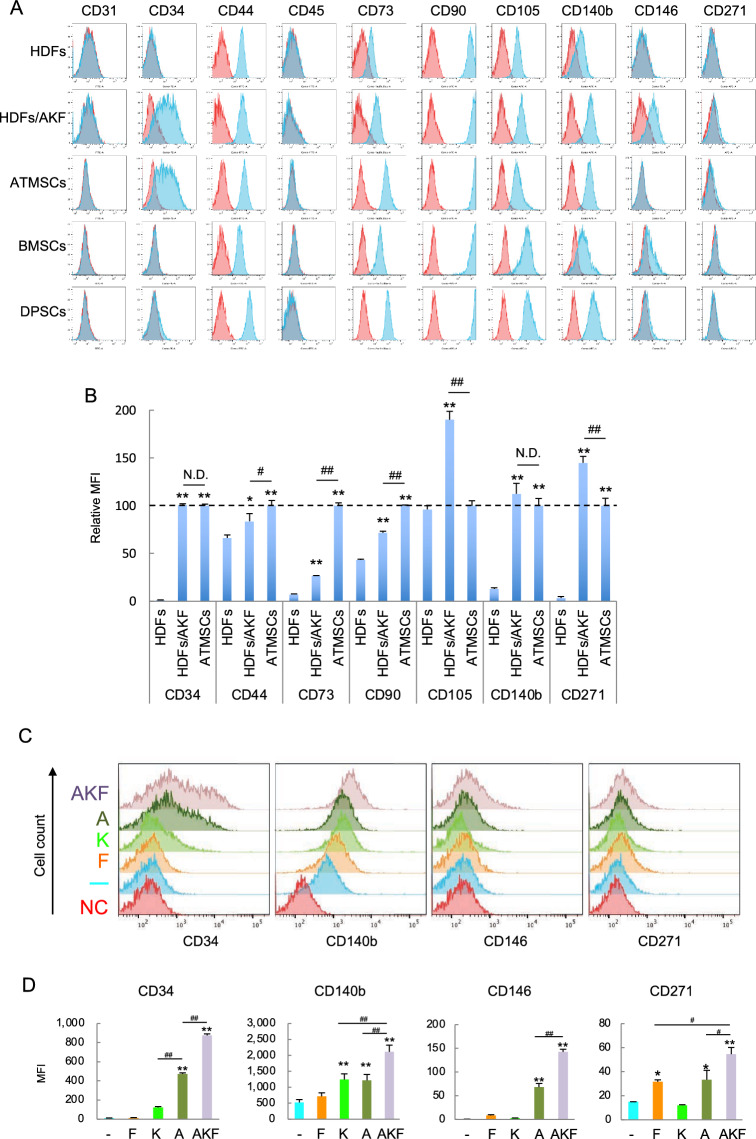
HDFs expressed MSC markers after treatment with ALK5iII (A), KU-60019 (K) and Fasudil (F) (AKF). **A** and **B** Untreated HDFs as a negative control, HDFs treated with AKF for 10 days (HDFs/AKF) and MSCs from various tissues as positive controls were immunostained with fluorescein-conjugated monoclonal antibodies. Histograms (A, blue lines) and mean fluorescence intensities (MFIs) (B) are shown. Red lines in (A) show staining with isotype-matched negative control antibodies (Ctrl Ab). **C** and **D** HDFs were treated with the indicated chemical compound(s) for 10 days, and subjected to flowcytometric analyses as above. Histograms (C) and MFIs (D) are shown. As negative control, HDFs were incubated with Ctrl Ab. Values in (B) and (D) are means ± S.D. n = 3. *P < *0.05* and **P < *0.01*, vs. the HDFs cultured without any compound. ^*#*^*P* < *0.05* and ^*##*^*P* < *0.01*, between the indicated groups

We treated the HDFs with a mixture of the three compounds, i.e., AKF and examined expression of cell surface markers. As shown by the histograms and the mean fluorescence intensities (MIFs), the resultant cells (HDFs/AKF) expressed typical MSC markers, CD44 antigen [Homing cell adhesion molecule (HCAM)], CD73 antigen (5′-nucleotidase), CD90 antigen [Thymocyte differentiation antigen 1 (Thy1)], CD105 antigen (Endoglin) and CD140b antigen [Platelet-derived growth factor receptor β (PDGFR-β)], at higher levels than original HDFs did (Fig. 1A–B). The AKF treatment also induced expression of other MSC markers, CD34 antigen, CD146 antigen [Melanoma cell adhesion molecule (MCAM)] and CD271 antigen [p75 neurotrophin receptor (p75NTR)], that HFDs did not express (Fig. 1A–B and Supplementary Figure S1H). These cell surface marker profiles of HDFs/AKF were basically similar to those of MSCs derived from three different tissues [adipose tissue-derived MSCs (ATMSC), bone marrow-derived MSCs (BMMSC) and dental pulp-derived MSCs (DPMSC)], although expression levels of the markers vary among MSCs of different origins.

We treated HDFs with each individual compound or various combinations and then compared the potential effects of the compounds to the expression of MSC markers in HDFs. We found that ALK5iII induced most MSC markers, while KU-60019 effectively up-regulated CD140b expression, and Fasudil induced CD271 expression (Fig. 1C–D). The combination of the three compounds (AKF) was considered most adequate for inducing high level expression of all the MSC markers in HDFs than a single compound or two compound combinations (Fig. 1C–D and Supplementary Figure S1I).

**Fig. 2:**
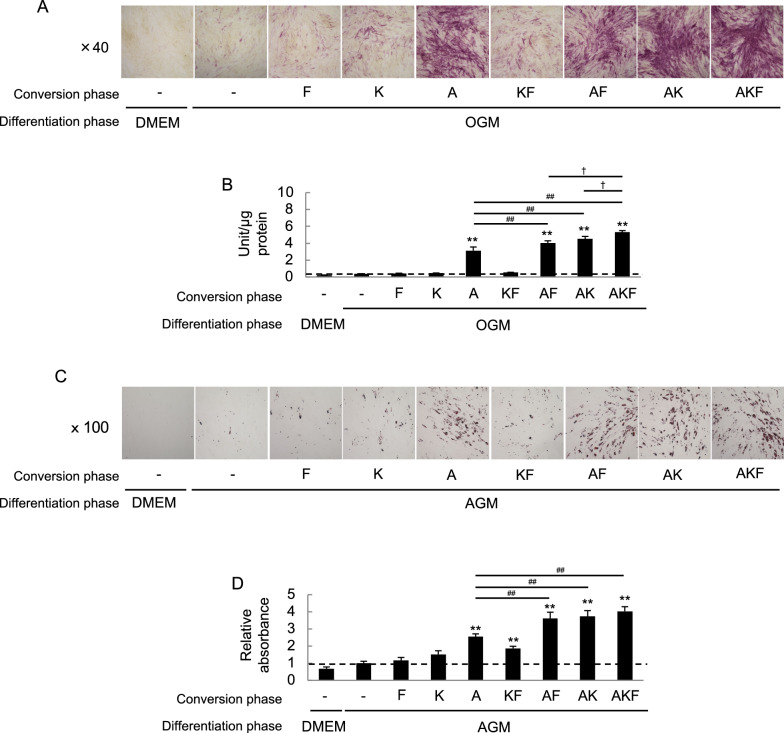
Osteogenic and adipogenic differentiation of HDFs treated with AKF. HDFs were treated with the indicated chemical compound(s) in CM to induce MSCs-like phenotypes (Conversion phase), followed by culture in either osteogenic medium (OGM) or adipogenic medium (AGM) to induce differentiation into osteoblasts or adipocytes (Differentiation phase). As indications of osteoblasts-like phenotypes, images of ALP staining (**A**) and ALP activities of the cell lysates (**B**) are shown. As indications of adipocyte-like phenotypes, microscopic images (**C**) and intensities (**D**) of Oil Red O staining are shown. Values in (**B**) and (**D**) are means ± S.D. n = 4. *P < 0.05 and **P < *0.01*, vs. the HDFs that were cultured without any compound in the conversion phase and cultured in OGM/AGM in the differentiation phase (second leftmost bars). ^*#*^*P* < *0.05* and ^*##*^*P* < *0.01* v.s. the HDFs that were cultured with A in the conversion phase and cultured in OGM/AGM in the differentiation phase (center bars). ^†^*P* < *0.05* and ^††^*P* < *0.01*, between the indicated groups

We estimated osteogenic and adipogenic differentiation potential of HDFs/AKF. HDFs were treated with A, K and/or F as above (Conversion phase), followed by culture in OGM or AGM (Differentiation phase). The HDFs treated with A, AF, AK or AKF showed high levels of ALP activities after culture in OGM (Fig. 2A and B). Culture under adipogenic conditions resulted in accumulation of lipid droplets in HDFs that were treated with A, KF, AF, AK or AKF (Fig. 2C and D). Among all the treatment recipes, the AKF treatment induced the strongest ability of multipotent differentiation to osteoblasts and adipocytes in HDFs (Figs. 2). We also checked effects of the AKF on HDFs from different donors namely HDF18 and HDF22. Both of them that were treated with the AKF expressed MSC markers at higher levels than original HDF18 and HDF22 (Supplementary Figure S2A). Based on these findings, the HDFs/AKF were brought to further detailed analyses as representative chemical compounds-driven directly converted MSCs (cdMSCs).

### cdMSCs showed ATMSC-like characters and functions in vitro

**Fig. 3:**
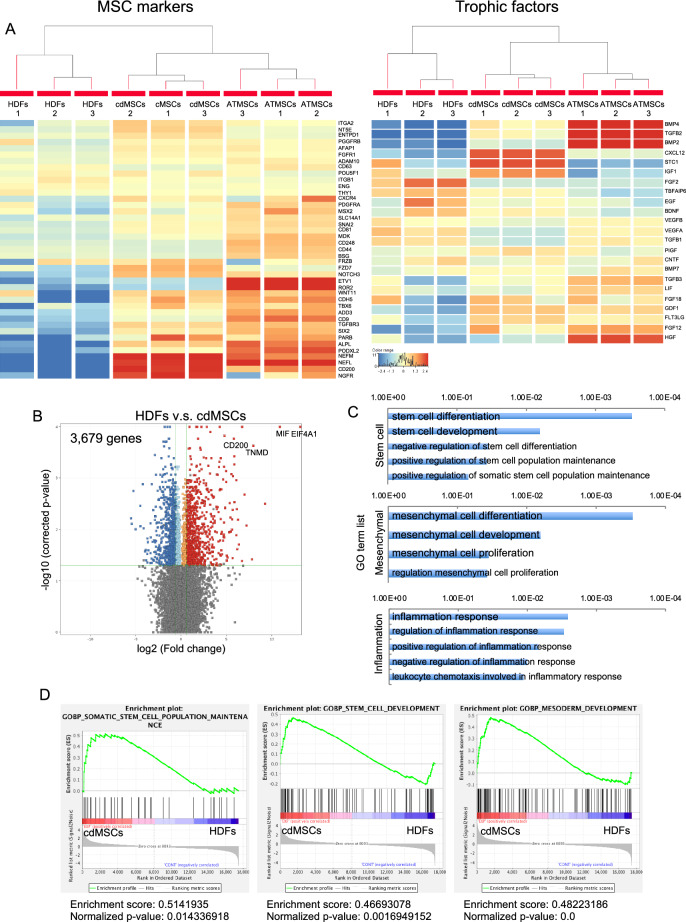
Transcriptome analyses confirmed genome-wide change in mRNA expression patterns during convertion from HDFs to cdMSCs. RNA was extracted from HDFs, cdMSCs converted from HDFs by AKF, and ATMSCs as a typical MSCs and subjected to RNA sequencing analysis. **A** Heat maps and hierarchical clustering data for MSC markers (Left) and trophic factor genes (Right). The genes with high expression are colored red, and those with low expression are colored blue as indicated in the color range. **B** Volcano plot. Red and blue dots represent genes for which mRNA levels were significantly different between HDFs and cdMSCs (3679 genes) (*P* < *0.05* and over 1.5-fold). **C** GO term analysis of the 3,679 genes for “stem cells”, “mesenchymal”, and “inflammation” categories. **D** Gene expression profiling of HDFs and cdMSCs. GSEA for the GO terms “Somatic Stem Cell Population Maintenance”, “Stem Cell Development”, and “Mesoderm Development”

**Fig. 4:**
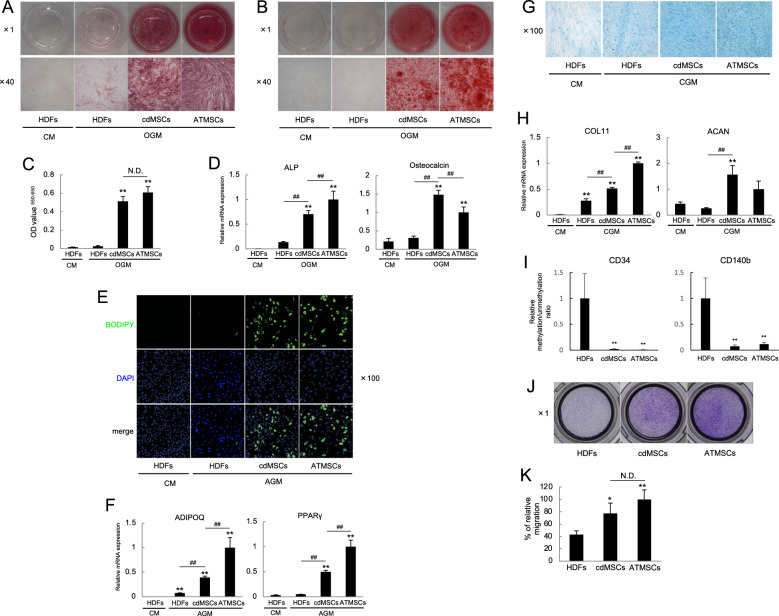
cdMSCs showed tri-lineage differentiation ability similar to ATMSCs. **A**–**D** HDFs as a negative control, cdMSCs converted from HDFs by AKF, and ATMSCs as a positive control were cultured in CM or OGM for 21 days. Osteoblast differentiation was visualized by ALP staining (**A**) and Alizarin Red S staining (**B**). Absorbance OD_550_–OD_650_ was determined to quantify the Intensity of Alizarin Red S staining (**C**), while relative mRNA levels of osteoblast-related genes were also measured (**D**). **E** and **F** Cells were cultured in CM or AGM for 21 days. Adipocyte differentiation was observed by staining with BODIPY and Hoechst 33429 (**E**), while relative mRNA levels of adipocyte-related genes were also examined (**F**). **G** and **H** Cells were cultured in CM or CGM for 21 days. Chondrocyte differentiation was estimated by Alcian blue staining (**G**), while relative mRNA levels of chondrocyte-related genes were also examined (**H**). **I** CpG DNA methylation statuses of CD34 and CD140b gene up-stream regions were analyzed, and relative methylation/unmethylation ratios are shown. **J** and **K** Migration ability of the cells was assessed by migration assay. Macroscopic images of the cells that migrated to bottom side of the upper chamber (**J**), and percentage of migrated cells (**K**) are shown. Magnifications were × 1 (Upper panels of A and B, and I), × 40 (Lower panels of A and B), and × 100 (E and G). Values are means ± S.D. n = 3 (C), and n = 4 (D, F, H and J). *P < *0.05* and **P < *0.01*, vs. the HDFs cultured in OGM, AGM or CGM. ^*#*^*P* < *0.05* and ^*##*^*P* < *0.01*, between the indicated groups. N.D., no significant difference between the indicated groups. **I** Values are means ± S.D. n = 4. **P < *0.01*, v.s. HDFs

To further characterize the cdMSCs, we estimated genome-wide gene expression profile of the cells by RNA sequencing analysis. We found that cdMSCs strongly expressed genes for MSC markers and trophic factors (Fig. 3A), and hierarchical clustering analysis revealed that the gene expression profiles of cdMSCs was more closely similar to ATMSCs than to original HDFs (Fig. 3A). Replicate analyses of all RNA sequencing data showed that expression levels of 3679 genes were significantly different between cdMSCs and HDFs. Among others, EIF4A1, MIF, TNMD and CD200 were the representatives that were strongly up-regulated in cdMSCs (Fig. 3B). The 3679 genes contained those annotated to the GO terms “stem cell development”, “stem cell population maintenance”, “mesenchymal cell development”, and “regulation of inflammatory response” were significantly affected by direct conversion (Fig. 3C). GSEA also clarified that the gene sets for “stem cell population maintenance”, “mesoderm development” and “mesodermal cell fate commitment” were significantly enriched in the cdMSCs in comparison with the original HDFs (Fig. 3D). In addition, pathway analysis based on the RNA sequencing data revealed that the direct conversion was accompanied with significant changes in expression levels of the genes related to TGF-β, MAPK, Hedgehog and WNT signaling pathways (Supplementary Figures S3). Next, we investigated whether cdMSCs have tri-lineage differentiation capacities by culturing them in OGM, AGM or chondrogenic medium (CGM). After culture in OGM, cdMSCs showed high ALP activity (Fig. 4A), production of calcified body matrix (Fig. 4B–C), and high expression of osteoblast-related genes (Fig. 4D). The cdMSCs cultured in AGM showed abundant lipid droplet formation (Fig. 4E) and highly expressed adipocyte-related genes (Fig. 4F). cdMSCs cultured in CGM produced glycosaminoglycans (Fig. 4G) and expressed chondrocyte-related genes at high levels (Fig. 4H). Similar results were obtained with cdMSCs derived from HDF18 and HDF22 (Supplementary Figures S2B–J).

These results indicated that cdMSCs have multipotency to differentiate into osteoblastic, adipogenic and chondrogenic lineages, which is similar to ATMSCs but not original HDFs.

Epigenetic statuses of HDFs, cdMSCs and ATMSCs were examined by checking CpG methylation on up-stream regions of CD34 and CD140b genes. Both regions were less methylated in cdMSCs and ATMSCs than in HDFs, strongly suggesting that the chemical compound-driven direct conversion is associated with epigenetic changes of chromosomal DNA (Fig. 4I).

We also investigated migration ability and anti-inflammatory function of cdMSCs in vitro. We found that cdMSCs had higher migration ability than original HDFs did (Fig. 4J and K), while cdMSCs significantly suppressed IL-1β production by THP-1 (Supplementary Fig. S2K). In addition, we checked MHC class ll expression of the cells, and confirmed that neither HDFs, cdMSCs, nor ATMSCs expressed HLA-DP, DQ, and DR, unlike THP-1 that expressed these molecules (Supplementary Fig. S2L). Thus, cdMSCs may lack antigen presentation ability, which is consistent with previous reports showing that MSCs do not present antigens [34, 35].

### cdMSC transplantation alleviated acute lung injury (ALI) of mice as significantly as ATMSCs did

**Fig. 5:**
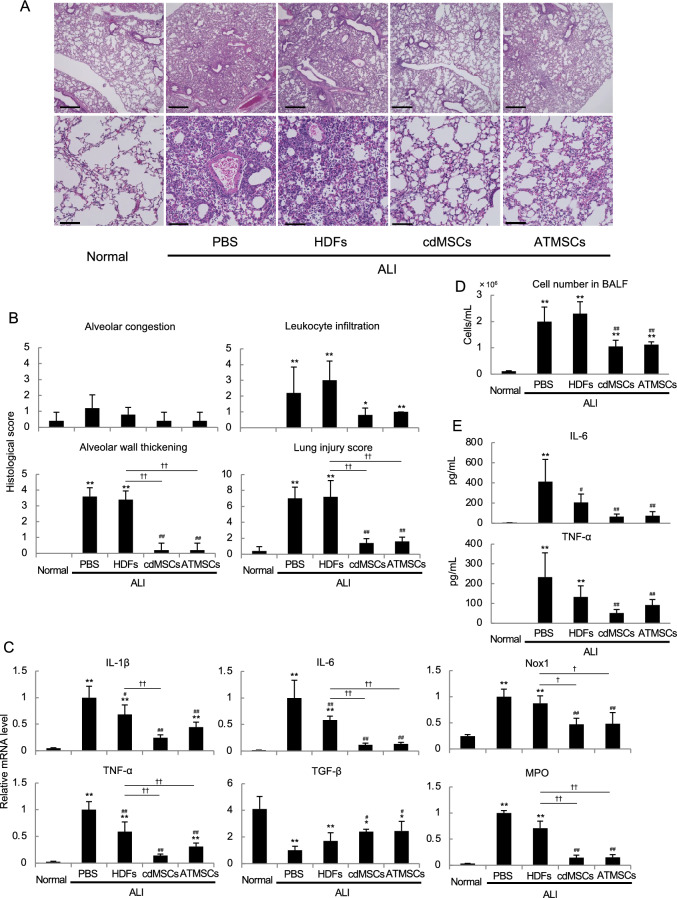
Transplantation of either cdMSCs or ATMSCs improved ALI in mice. NOG mice were treated with LPS to induce ALI, followed by transplantation with HDFs, cdMSCs or ATMSCs. Negative control mice were not given LPS (Normal), while mock-treated ALI mice were administered with PBS instead of cell transplantation (PBS). **A** Microscopic images of H & E-stained lung tissue Sections 48 h after LPS instillation. Scale bar = 500, and 100  μm. **B** Degrees of lung injury was evaluated by semi-quantitative scoring as described in the Materials and Methods. **C** Relative mRNA levels for the indicated genes in lung tissue. **D** and **E** Cell numbers (**D**) and cytokine concentrations (**E**) in BALF. Values in **B** to **E** are means ± S.D. n = 5. *P < *0.05* and **P < *0.01*, vs. Normal group. ^*#*^*P* < *0.05* and ^*##*^*P* < *0.01*, vs. PBS group. ^†^*P* < *0.05* and ^††^*P* < *0.01*, between the indicated groups

Next, we examined functions in vivo of the cdMSCs using two types of disease models in mice, both of which have been used for the evaluation of MSCs’ function in vivo in previous reports [32, 36, 37]. First, we applied HDFs, cdMSCs and ATMSCs to ALI model mice, because massive bacterial LPS instillation manifest acute inflammation in the lung characterized by patchy intra-alveolar polymorphonuclear leukocyte infiltration and a mild increase in epithelial permeability at acute stages. Mice in all groups survived during the experiment, but we found that cdMSC- and ATMSC-transplanted mice showed significantly less histological evidence of injury in the lung tissue than PBS- and HDF-given mice did (Fig. 5A and B). PBS-administrated mice and HDF-transplanted mice showed conspicuous leukocyte infiltration and alveolar wall thickening in their lungs (Fig. 5A and B). In contrast, lung tissue of cdMSC- and ATMSC-transplanted mice didn’t exhibit such apparent signs of lung inflammation (Fig. 5A and B). mRNA for inflammatory cytokines, IL-1β, IL-6 and TNF-α, and oxidative stress indicators, Nox1 and MPO, was significantly elevated in the lung tissue in PBS- and HDF-given mice (Fig. 5C), but less significantly increased in the lung tissue of cdMSC- and ATMSC-transplanted mice (Fig. 5C). In contrast, TGF-β gene mRNA was highly expressed in cdMSC- and ATMSC-transplanted mice than in PBS-administrated mice (Fig. 5C). Examination of bronchoalveolar lavage fluid (BALF) also revealed that the cell number as well as concentrations of IL-6 and TNF-α were significantly lower in the ALI mice transplanted with cdMSCs or ATMSCs than in the PBS-applied ALI mice (Fig. 5D and E). These results suggest that cdMSCs can suppress lung injury in this disease model as strongly as ATMSCs do through their anti-inflammatory functions.

### cdMSC transplantation significantly relieved chronic autoimmune arthritis in mice

**Fig. 6:**
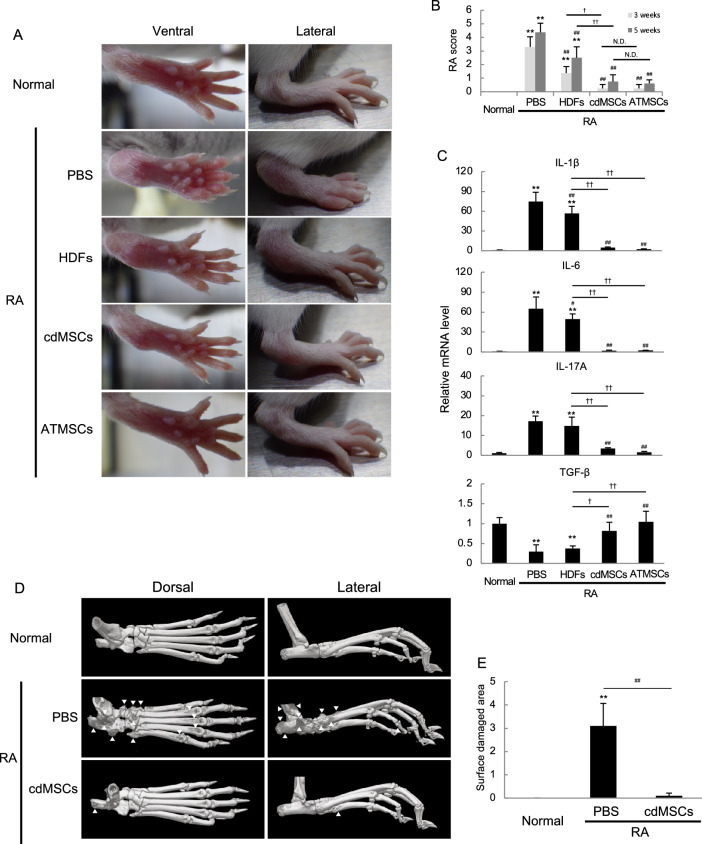

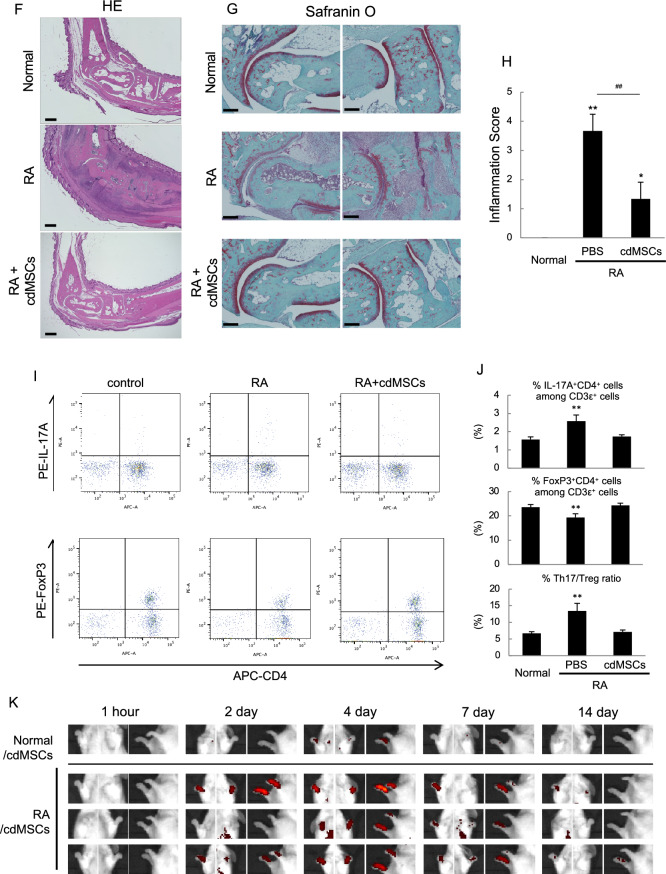
Transplantation of either cdMSCs or ATMSCs ameliorated autoimmune arthritis in mice. SKG/Jcl mice were administered with laminarin to induce RA-like joint inflammation, followed by transplantation with HDFs, cdMSCs or ATMSCs. Negative control mice were not given laminarin (Normal), while mock-treated RA mice were administered with PBS instead of cell transplantation (PBS). **A** Representative macroscopic images of hind paws at 5 weeks. **B** RA scores for mice at 3 and 5 weeks after administration with laminarin. **C** Relative mRNA levels for cytokine genes in paw tissues of forelimbs of mice at 5 weeks. **D** Representative micro-CT images of ankles of hindlimbs of mice at 5 weeks. **E** Areas of damaged bone surface evaluated by micro-CT examination. **F** and **G** Microscopic images of hindlimb ankle sections stained with H & E (F) and Safranin O (G) at 5 weeks. Scale bars = 500 μm (F) and 200 μm (G). **H** Histological inflammation scores at 5 weeks. **I** and **J** Splenocytes were obtained from the indicated mice at 5 weeks, and analyzed by flowcytometry. Gated-CD3ε-positive splenocytes are displayed in dot plots for CD4 vs. either anti-IL17A or anti-FoxP3 staining (I). Values in (I) show mean percentages of cells in the quadrants. Percentages of IL-17A + CD4 + cells and FoxP3 + CD4 + cells among CD3ε + cells as well as Th17/Treg ratios are also shown (J). **K** cdMSCs were labelled with Dil and transplanted to negative control or mock-treated RA mice. Representative chronological IVIS images are shown. Values in (B, C, E, H, J) are means ± S.D. n = 4 (B, C, J) or 3 (E, H). *P < *0.05* and **P < *0.01*, vs. Normal group. ^##^*P* < *0.01*, vs. PBS group. ^†^*P* < *0.05* and ^††^*P* < *0.01*, between the indicated groups

To figure out if cdMSCs are also therapeutically useful for an inflammatory disease associated with autoimmunity, we used SKG /Jcl mice that develop T cell-mediated chronic autoimmune arthritis resembling human rheumatoid arthritis (RA) in terms of the symptoms and molecular immunopathogenesis [31]. Macroscopically, apparent joint swelling and redness were observed in PBS- and HDF-applied RA mice, and the symptoms were exacerbated between 3 and 5 weeks after arthritis induction as demonstrated by RA scores for the mice (Fig. 6A and B). However, RA score was significantly reduced in cdMSC- and ATMSC-transplanted mice both 3 and 5 weeks after arthritis induction (Fig. 6A and B). We also evaluated cytokine expression status using forelimb tissue. IL-1β, IL-6 and IL-17 mRNA was significantly increased in PBS- and HDF-applied RA mice, but the expression was significantly suppressed in cdMSC- and ATMSC-treated RA mice (Fig. 4F). In contrast, TGF-β mRNA expression in cdMSC- and ATMSC-treated RA mice were as high as that in the normal (non-RA) mice (Fig. 6C).

For further estimation of the anti-inflammatory effects, hind limbs of control, RA and cdMSC-treated RA mice were subjected to μCT examination and histological analysis. The μCT images demonstrated apparent bone destruction at the surface of ankle joint in non-transplanted RA mice, whereas such bone destruction was hardly seen in the normal (non-RA) and cdMSC-treated RA mice (Fig. 6D and E). Histologically, any remarkable sign of inflammation was not found at the joint tissue of the non-RA mice, but RA mice manifested many pathological indications such as bone and cartilage erosion, moderate to severe thickening of ankle tissue, and massive immune cell infiltrating (Fig. 6F and G). While, the histology of cdMSC-transplanted mice showed slight thickening of the joint tissue, and/or limited immune cell infiltration, so that their inflammation score was significantly lower than that of RA mice (Fig. 6H). We also assessed the proportions of Treg and Th17 in splenocytes from these mice. The splenocytes harvested from non-transplanted RA mice contained lower percentage of FoxP3 + CD4 + cells than those from cdMSC-transplanted RA mice and non-RA control mice (Fig. 6I and J). On the other hand, non-transplanted RA mice had higher percentage of IL-17A + CD4 + cells in the spleen than the cdMSC-treated RA mice and non-RA control mice (Fig. 6I and J). The Th17/Treg ratio of cdMSC-treated RA mice was comparable to non-RA control mice (Fig. 6J). Furthermore, we checked distribution of cdMSCs after transplantation into the RA model mice by in vivo imaging. DiR-labeled cdMSCs migrated and homed to the inflammatory lesions in the limbs of RA mice 2 day after transplantation, and localized there for more than 7 days, before they disappeared at 14 days (Figs. 6I and Supplementary Figure S4). Such localization of cdMSCs in limbs was not evident in non-RA mice (Fig. 6K). These results indicate that cdMSCs can home to the inflammatory lesions and ameliorate inflammatory status related to autoimmune diseases.

### Exosomes secreted by cdMSCs suppressed M1 macrophage induction while enhancing M2 macrophage induction

**Fig. 7:**
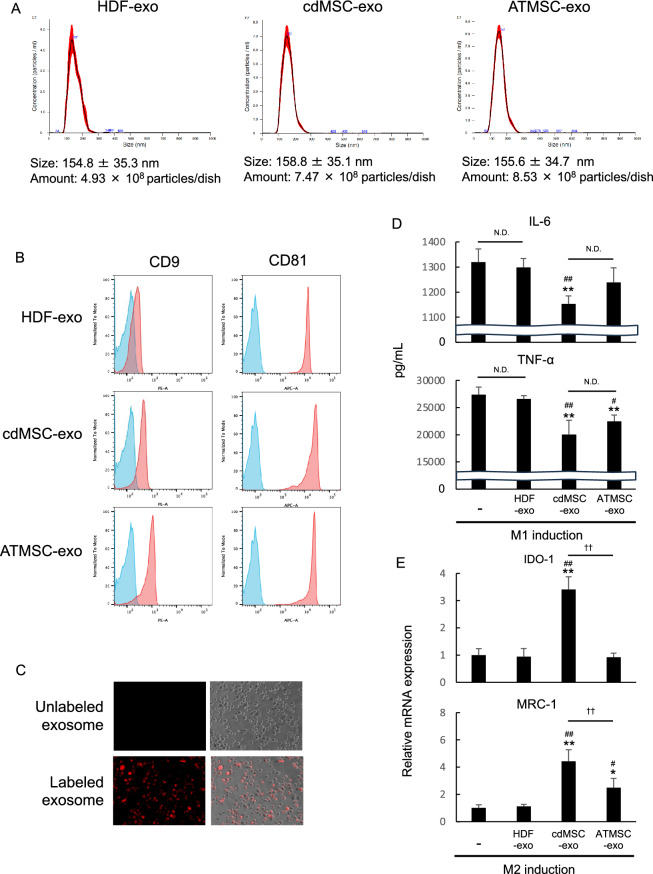
cdMSC-exo suppressed M1 polarization and enhanced M2 polarization of macrophages. **A** HDF-exo, cdMSC-exo, and ATMSC-exo were isolated and analyzed by nanoparticle tracking assessment (NTA). Size distribution and yield of the particles are shown. **B** Exosomes were tested for the expression of exosomal markers by flowcytometry. Light blue histograms show the fluorescent signals of the beads alone control samples without exosomes. **C** M0 macrophages were cultured with un-labelled cdMSC-exo or cdMSC-exo labelled with a fluorescent dye for 24 h. Fluorescence microscopic images are shown. Magnification was × 100. **D** Concentrations of IL-6 and TNF-α (M1 markers) produced from macrophages cultured under M1 polarization condition in the presence/absence of the indicated exosomes. **E** mRNA levels for IDO-1 and MRC-1 genes (M2 markers) in macrophages cultured under M2 polarization condition in the presence/absence of the indicated exosomes. Values in (D and E) are means ± S.D. n = 4 *P < *0.05* and **P < *0.01*, vs. without exosome group. ^#^*P* < *0.05 and* ^##^*P* < *0.01*, vs. HDF-exo group. ^††^*P* < *0.01*, between the indicated groups

It has been reported that exosomes derived from MSCs have anti-inflammatory function as MSCs do [38]. To elucidate the mechanisms through which the cdMSCs suppressed inflammation, we isolated exosomes from the culture supernatants of HDFs, cdMSCs, and ATMSCs (HDF-exo, cdMSC-exo, and ATMSC-exo, respectively), and estimated their effects on macrophage differentiation in vitro. The nanoparticle tracking analysis (NTA) demonstrated that all the exosome samples had similar diameters in the range of 100 – 200 nm regardless of their cell sources, while greater numbers of the cdMSCs-exo and ATMSC-exo were yielded from the same volume of the culture supernatants compared with the HDF-exo (Fig. 7A). Flowcytometric analysis showed that all the exosome samples expressed tetraspanins CD9 and CD81, although their expression levels were different depending on the cells by which the exosomes were secreted (Fig. 7B). A human monocytic cell line, THP-1, were allowed to differentiate to M0 macrophages. An addition of fluorescence-labeled exosomes resulted in significant uptake of the exosomes by the M0 macrophages (Fig. 7C). The M0 macrophages were induced to M1 or M2 macrophages in the presence or absence of the exosomes. The cdMSC-exo significantly suppressed IL-6 and TNF-α production by the macrophages under the M1-inducing condition, while significantly augmenting the expression of MRC-1 and IDO-1 under the M2-inducing condition (Fig. 7D). Such effects were not exerted by the HDF-exo. These results indicate the possibility that cdMSC-exo may, at least partly, contribute to the suppression of acute and chronic inflammation by cdMSCs transplantation.

## Discussion

In the present study, we accomplished induction of MSC-like characters in HDFs by simply culturing the cells in the presence of a cocktail of three small chemical compounds without exogenous gene transduction or addition of any cytokine. The MSC-like cells (cdMSCs) showed similar phenotypes to ATMSCs in terms of gene expression profiles, immunophenotypes and differentiation potentials in vitro, as well as therapeutic usefulness for two different types of inflammatory diseases in vivo.

Direct conversion to MSCs from easily accessible somatic cells like fibroblasts could be one of promising ways to overcome the current problems in autologous MSC preparation for regenerative therapies. It has been reported that vascular wall typical MSC-like cells (VW-MSCs) were directly induced from fibroblasts by transducing VW-MSC-specific transcription factor genes, HOXB7, HOXC6 and HOXC8 [39]. However, unlike the vwMSCs, specific master regulator(s) for MSC differentiation have not been identified, making it difficult to induce MSC-like cells from other somatic cells by gene transduction. Besides, Lai et al. reported that BMMSC-like cells were induced in fibroblasts by culturing them with a specific cocktail of six compounds and three growth factors (6C + 3GF) [40]. The six compounds that they used and our three compounds are different, except for the ROCK inhibitor. More importantly, we need not to add any growth factors for the cell conversion, whereas LIF, TGF-β, and bFGF are required for the procedure by Lai et al.

Our cdMSCs expressed not only typical MSC markers like CD44, CD73, CD90 and CD105 but also CD34, CD146 and CD271. In consistent with previous reports [41], some of MSC markers were also detected in original HDFs, but the expression of CD73 and CD140b was significantly up-regulated in cdMSCs than in HDFs. It has been reported that high CD73 expression in MSCs was associated with high anti-inflammation activities and strong tissue repairing capabilities [42], while CD140b expression in MSCs was positively correlated with their multi-potent differentiation, and migration abilities [43]. CD34, CD146 and CD271 were expressed in cdMSCs but not original HDFs. CD34 was previously known as a negative marker for MSCs, but several recently articles reported that some types of MSCs expressed CD34 at low passages [44, 45]. In addition, it has been reported that CD34 is expressed on the surface of MSCs progenitors and MSCs in early stage [46, 47]. Thus, our cdMSCs seems to be embryonic ones. Also, expression of CD34 was correlated with angiogenic and high tissue regeneration capabilities [48, 49]. Although CD146 was not detected in ATMSCs, BMSCs and DPSCs expressed CD146 (Fig. 1A), and it was reported that the high expression levels of CD146 are positively correlated with therapeutic potentials of MSCs [50, 51]. So far, CD271 has been considered as one of the most reliable markers for definition of MSC characters and their therapeutic potential [52]. CD90 and CD271 double positive MSCs showed the highest proliferation, migration and differentiation abilities [53].

RNA sequencing analyses revealed that 3679 genes were significantly changed in expression levels by AKF treatment (over 1.5-fold and *P* < 0.05), although differential effect of each single compound has not been clarified. Among them, eukaryotic initiation factor 4A (eIF4A1), macrophage migration inhibitory factor (MIF), tenomodulin (TNMD), and CD200 were strongly up-regulated in AKF-treated cells (Fig. 3B). It has been reported that MIF and TNMD could inhibit stem cell senescence and prolong MSC survival [54–56]. eIF4A1 is essentially involved in Cap-dependent protein translation, and present in the micro-vesicles produced from MSCs [57]. CD200, a membrane of the immunoglobulin superfamily, was recently identified as one of the novel markers for native MSCs. And it has been reported that CD200-positive MSCs exert high immune modulatory and anti-inflammatory functions through CD200/CD200R interaction [58]. Moreover, gene sets related to stem cells and inflammation were drastically changed in expression levels by the AKF treatment (Fig. 3C) as revealed by GO term analysis.

ALK5iII inhibits TGF-β receptor-1/actin like receptor-5 (TGF-β R-1/ALK5) signaling. TGF-β pathway plays important roles in several cellular activities such as proliferation, senescence and differentiation [59]. TGF-β R inhibitors enhance expression of pluripotent markers such as Oct4, Sox2 and Nanog during iPS cell induction [60, 61]. They are essential for chemical direct conversion of fibroblasts to cardiomyocytes [62], neurons [63], and osteoblasts [30]. Blockage of TGF-β R signaling is associated with suppression of profibrotic signals. Also, with regards to the role of TGF-β on MSCs, TGF-β accelerates cellular senescence of MSCs [64], while a TGF-β R inhibitor improves induction of MSC characters by up-regulating immunophenotypes and multi-differentiation abilities of MSCs [65, 66]. Fasudil is a selective ROCK inhibitor that prevents stem cell apoptosis, activates chromatin-remodeling genes responsible for epigenetic modification, and is involved in maintenance of stem cell characters [67, 68]. KU-60019 is an inhibitor of the serine/threonine kinase, ataxia telangiectasia mutated (ATM), that plays essential roles in the maintenance of genomic stability. A previous report indicated that ATM inhibition by KU-60019 alleviated cellular senescence of aged fibroblasts [69]. Also, co-inhibition of ROCK and ATM synergistically enhanced cell proliferation and decelerated cellular senescence [70]. Our findings that the AKF treatment induced MSC-like phenotypes in HDFs could be explained by these effects of the three inhibitors. Moreover, it was also reported that inhibition of TGF-β-smad cooperates with WNT and MAPK activation to potentiate stem cell functions [71, 72]. Actually, expression of the genes related to TGF-β, MAPK, Hedgehog and WNT signaling pathways was broadly and substantially affected by the AKF treatment (Supplementary Figures S3).

For estimation of cdMSC function in vivo, we chose ALI and SKG arthritis as the disease models. The former is an LPS-induced acute inflammation model, while the latter is a chronic inflammation model caused by autoimmunity, and both responded to MSC transplantation in previous literatures [32, 36, 37]. In the SKG arthritis model, it was reported that excess production of IL-6 and IL-17 and imbalance between Th17 and Treg in articular tissue are critically involved in the immunopathogenesis of SKG arthritis [73]. Thus, we evaluated cytokine expression in articular tissue and proportion of Th17 and T reg in splenocytes. We found that the expression levels of IL-6, IL-17 and other inflammatory cytokines were significantly increased in the articular tissue of RA group, while the expression level was decreased in cdMSC- and ATMSC-transplanted groups. This is consistent with a previous report in which ATMSCs were transplanted to SKG mice [32]. In addition, we found that the proportion of splenic Treg was reduced in the RA group but not in the groups given transplantation of either cdMSCs or ATMSCs. These may at least partially explain the mechanism of the anti-rheumatic effects of MSC transplantation. Transplantation of cdMSCs prevented aggravation of both acute and chronic model diseases as ATMSC transplantation did, indicating that cdMSCs can work not only in vitro but also in vivo.

Recently, it has been reported that an administration of the exosomes derived from MSCs has prominent potential comparable to MSC transplantation in regenerative therapy [38]. The exosomes may contain growth factors, signaling lipids, mRNAs and regulatory miRNAs. Use of the exosomes may bring a paradigm shift from cell transplantation to cell free therapy. Some clinical trials have revealed significant therapeutic outcomes of MSC-exo administration to patients with inflammatory diseases [74]. In this study, we found that cdMSCs produced as a high number of exosomes as ATMSCs. To our best knowledge, this is the first report in which exosomes secreted from the directly converted MSCs were isolated. Also, we confirmed that the cdMSC-exo suppressed M1 macrophage polarization (Fig. 7D), while enhancing induction of M2 macrophage phenotype (Fig. 7E). Although further investigation is required, our findings strongly suggest that the exosomes derived from cdMSCs are a quite simple, safe, economical and promising therapeutic modality for regenerative medicine.

Taken together, this study may have a significant implication for understanding the epigenetic mechanisms of cell fate conversion, and propose novel promising therapeutic options for inflammatory diseases by providing MSCs that can be relatively easily and safely prepared from autologous fibroblasts and are suitable for transplantation.

## Conclusion

Specific combination of three compounds (AKF), i.e., a TGF-β receptor inhibitor, ROCK inhibitor and ATM inhibitor, provoked HDFs to strongly express MSC markers, and the resultant cdMSCs showed osteogenic, adipogenic and chondrogenic differentiation potentials. The phenotype conversion was associated with epigenetic changes of chromosomal DNA. The cdMSCs also relieved inflammatory responses in the mice with acute and chronic inflammatory diseases. Furthermore, the cdMSC produce exosome and the exosome (cdMSC-exo) suppress M1 macrophage induction, while enhance M2 macrophage induction. These results may propose a solution to overcome the limitation of MSCs obtained from bone marrow or adipose tissue of patients and pave the way to the use of cdMSCs for regenerative therapy and cell transplantation therapy.

## Supplementary Information

Additional file 1 (DOCX 19 KB)

Additional file 2 (PDF 1970 KB)

## Data Availability

All data associated with this study are present in the main text or the supplementary information. All RNA sequencing data for this study have been deposited in NCBI’s Gene Expression Omnibus (GEO) database under accession number GSE25005.
