# Supplementary material for: Chemical direct conversion of human fibroblasts to mesenchymal stem cells that can alleviate inflammation in vivo
Source: Stem Cell Res Ther. 2025 Oct 30;16:597. doi: 10.1186/s13287-025-04605-x (PMC12574161; doi:10.1186/s13287-025-04605-x)
Supplement: Supplementary file 1 — Additional file 1 (DOCX 19 KB) [file 13287_2025_4605_MOESM1_ESM.docx]

**Supplementary Figures legend**

**Fig. S1:** Chemical induction of osteogenic and adipogenic differentiation capacities and MSC markers in HDFs.

A-C, HDF45 cells were cultured in OGM supplemented with various concentrations of ALK5iII. Microscopic images of ALP staining at 10 days (A) and Alizarin Red S staining at 13 days (B) are shown (magnification, x 40). Intensities of Alizarin Red S staining are also shown (C). D and E, HDF45 were cultured in AGM with various concentrations of ALK5iII for 13 days. Fluorescence microscopic images of BODIPY plus Hoechst 33429 staining (magnification,x 100) (D) and relative mRNA levels for adipogenic genes (E) are shown. F and G, HDF45 were cultured in CM, OGM or AGM in the presence of Fasudil (F) or KU-60019 (K) for 21 days. Images of ALP staining (magnifications, x 1 and x 40) (F) and fluorescence microscopic images of BODIPY and Hoechst 33429 staining (magnification, x 100) (G) are shown. H, Relative MFI of CD146 for HDFs, HDFs treated with AKF for 10 days (HDFs/AKF) and BMSCs. I, HDFs were treated with various combinations of ALK5iII (A), Fasudil (F) and KU-60019 (K) for 10 days, followed by flowcytometric analysis using the indicated fluorescence-labeled antibodies. Histograms are shown. NC represents cells stained with an isotype-matched negative control antibody. Values in (C) are means ± S.D., n = 3. **P < 0.05* and ***P < 0.01*, v.s. OGM. *^##^P < 0.01* between the indicated each groups. Values in (E) are means ± S.D., n = 3. *^##^P < 0.01* between the indicated each groups. Values in (H) are means ± S.D. n = 3. ***P < 0.01*, vs. the HDFs cultured without any compound.

**Fig. S2:** AKF treatment induced MSC-like phenotypes in HDFs from different donors.

(A) Two HDF cells line (HDF18 and HDF22) were treated with AKF for 10 days and immunostained with the indicated antibodies (blue histograms). Red histograms represent cells stained with an isotype-matched negative control antibody. (B) HDF45, HDF18 and HDF22 cells were treated with AKF. RNA extraction and RNA sequencing analyses were performed as in Fig. 2. Heat map and hierarchical clustering analyses of mRNA levels for MSC-related genes and trophic factor genes are shown. The genes with high expression are colored red, while those with low expression are colored blue. C-J, HDF18 and HDF22 cells were treated with AKF for 10 days to obtain HDF18/AKF and HDF22/AKF. The cells were cultured in CM, OGM, AGM and CGM for 21 days. Images of ALP staining and ALP mRNA expression levels (C and D), microscopic images and OD_550_ values of Alizarin Red S staining (E and F), fluorescence microscopic images of BODIPY and Hoechst 33429 staining and PPARγ mRNA levels (G and H), and microscopic images of Alcian blue staining and COL11 mRNA levels (I and J) are shown. Magnification of the images are x1 (C-F, upper panels), x 40 (C-F, lower panels), x 100 (G-J). Values are means ± S.D. n = 4. **P < 0.05* and ***P < 0.01*, v.s. CM. *^##^P < 0.01*, between the indicated groups. K, IL-1β concentrations in the supernatants of the indicated cell culture with/without LPS. Values are means ± S.D. n = 3. **P < 0.05* and ***P < 0.01*, vs. THP-1. *^#^P < 0.05* and *^##^P < 0.01*, vs. THP-1/LPS group. L, Cells were stained with APC-labeled anti-HLA-DP, DQ, and DR antibody and analyzed by flowcytometry. Histograms are shown.

**Fig. S3:** Genes differentially expressed by HDFs and cdMSCs may be involved in the TGF-b, WNT, MAPK and Hedgehog pathways.

Pathway analysis was performed for the genes of which mRNA expression was altered by the AKF treatment (Fig. 3A). The differentially expressed genes in the indicated pathways are highlighted in orange box. The square immediately to the right of each orange box represents expression level of the gene in HDF45, and the one further to the right represents expression level in cdMSC45. The red, yellow, and blue squares indicate high, moderate and low expression, respectively.

**Fig. S4:** Whole body IVIS images of ctrl and RA mice transplanted with cdMSC

Chronological IVIS images of the indicated mice are shown. Yellow, red and dark red coloring represent high, intermediate, and low fluorescence signals, respectively.
