# Supplementary material for: Chemical direct conversion of human fibroblasts to mesenchymal stem cells that can alleviate inflammation in vivo
Source: Stem Cell Res Ther. 2025 Oct 30;16:597. doi: 10.1186/s13287-025-04605-x (PMC12574161; doi:10.1186/s13287-025-04605-x)
Supplement: Supplementary file 2 — Additional file 2 (PDF 1970 KB) [file 13287_2025_4605_MOESM2_ESM.pdf]

Fig. S1

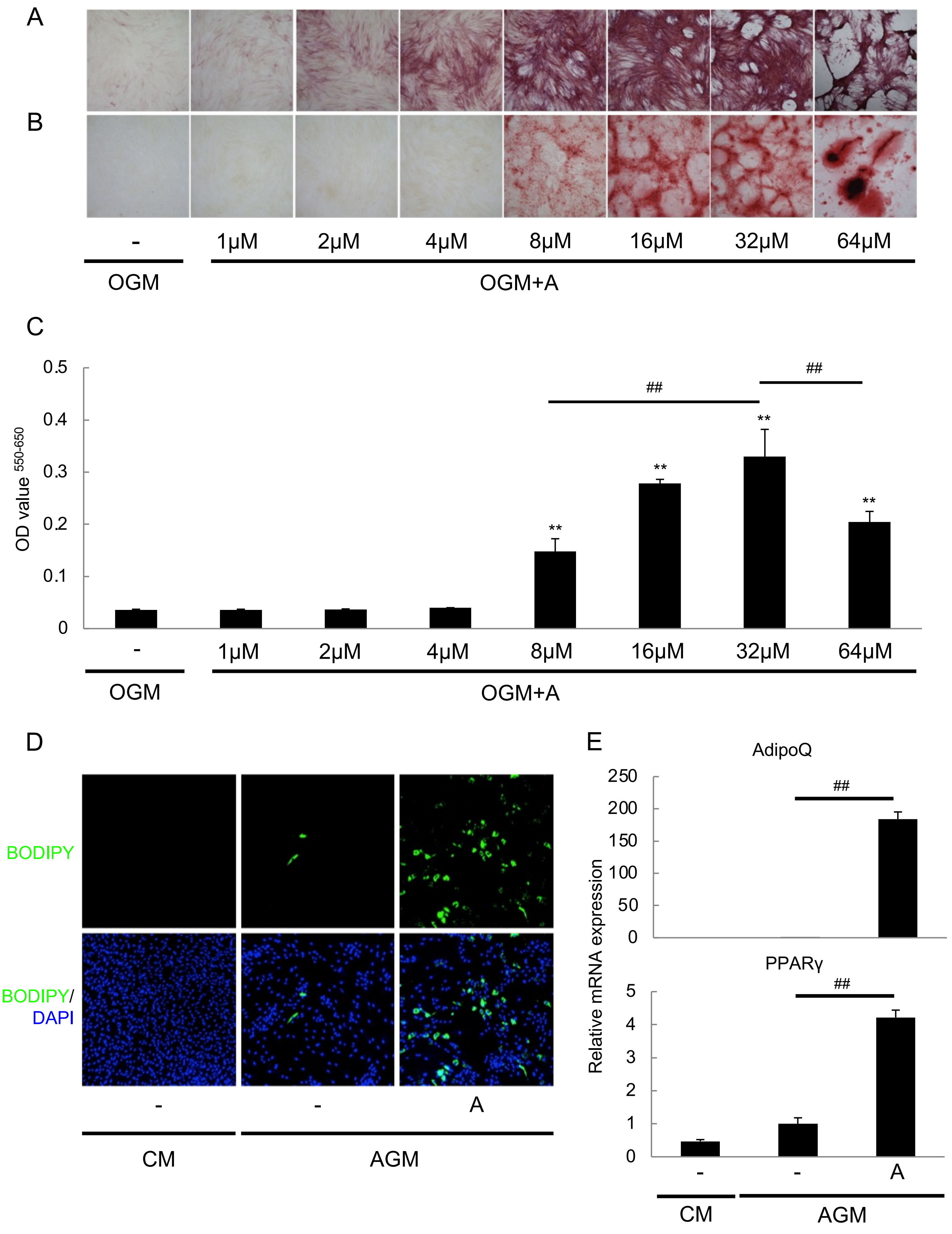

A
B
-
1μM
2μM
4μM
8μM
16μM
32μM
64μM
OGM
OGM+A
C
OD value 550-650
0.5
0.4
0.3
0.2
0.1
0
##
##
**
**
**
**
-
1μM
2μM
4μM
8μM
16μM
32μM
64μM
OGM
OGM+A
D
BODIPY
BODIPY/
DAPI
-
-
A
CM
AGM
E
AdipoQ
250
200
150
100
50
0
##
Relative mRNA expression
PPARγ
5
4
3
2
1
0
##
-
-
A
CM
AGM

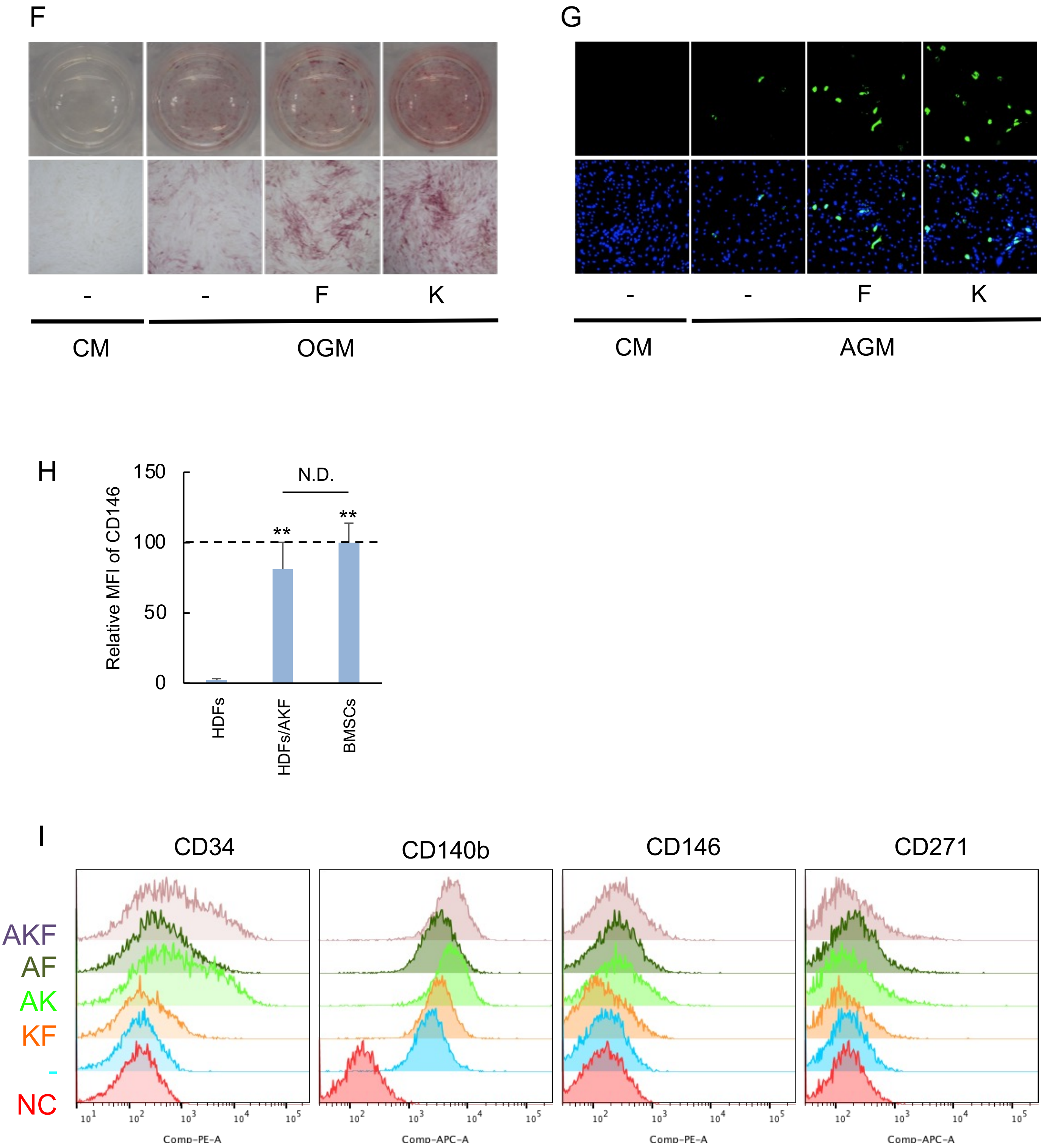
F
-
-
F
K
CM
OGM
G
-
-
F
K
CM
AGM
H
150
100
50
0
Relative MFI of CD146
N.D.
**
**
HDFs
HDFs/AKF
BMSCs
I
CD34
CD140b
CD146
CD271
AKF
AF
AK
KF
-
NC
Comp-PE-A
Comp-APC-A
Comp-PE-A
Comp-APC-A

Fig. S2

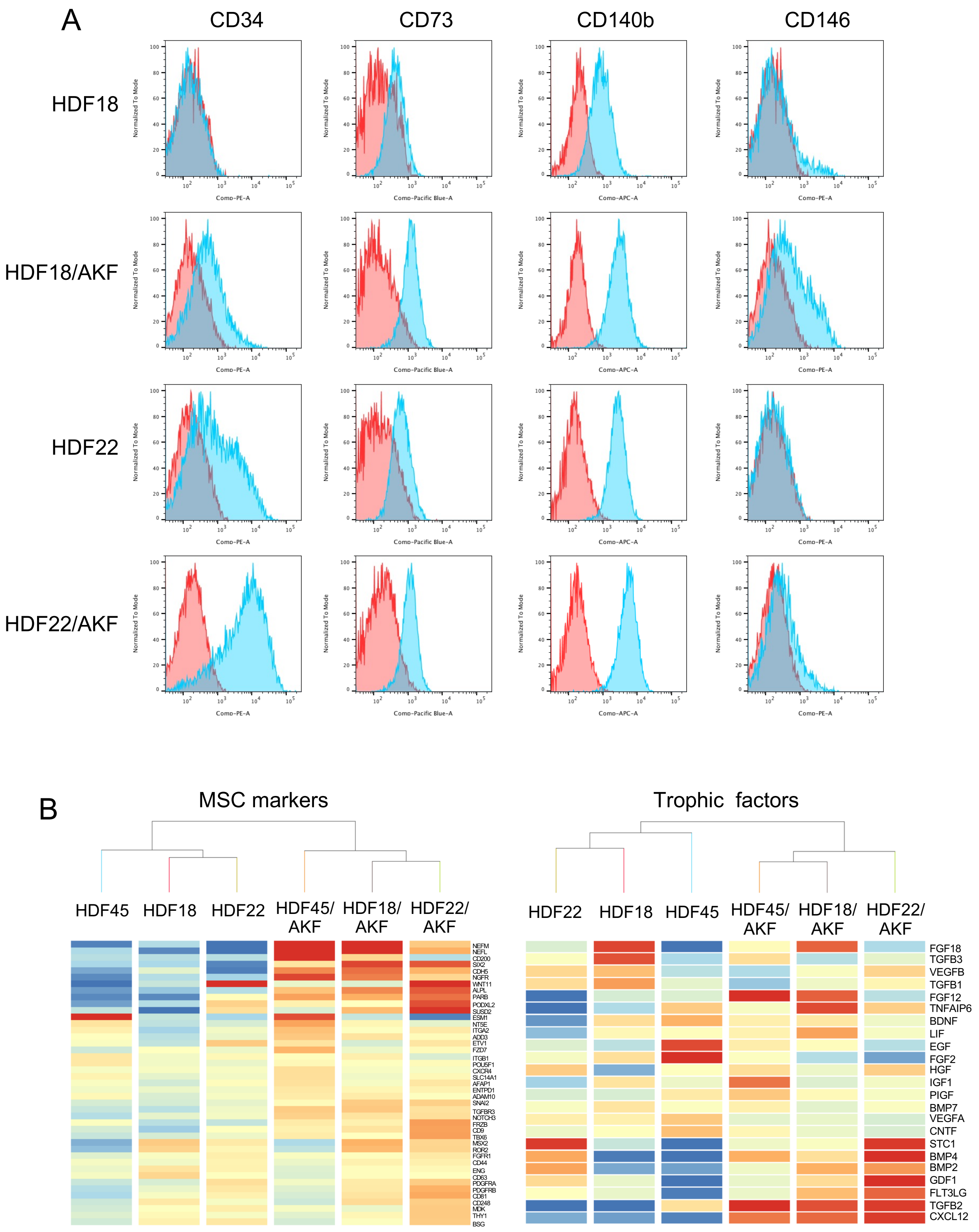
A
CD34
CD73
CD140b
CD146
HDF18
HDF18/AKF
HDF22
HDF22/AKF
Normalized To Mode
Comp-PE-A
Comp-Pacific Blue-A
Comp-APC-A
B
MSC markers
Trophic factors
HDF45
HDF18
HDF22
HDF45/ AKF
HDF18/ AKF
HDF22/ AKF
NEFM
NEFL
CD200
SIX2
CDH5
NGFR
WNT11
ALPL
PARB
PODXL2
SUSD2
ESM1
NT5E
ITGA2
ADD3
ETV1
FZD7
ITGB1
POU5F1
CXCR4
SLC14A1
AFAP1
ENTPD1
ADAM10
SNAI2
TGFBR3
NOTCH3
FRZB
CD9
TBX6
MSX2
ROR2
FGFR1
CD44
ENG
CD63
PDGFRA
PDGFRB
CD81
CD248
MDK
THY1
BSG
FGF18
TGFB3
VEGFB
TGFB1
FGF12
TNFAIP6
BDNF
LIF
EGF
FGF2
HGF
IGF1
PIGF
BMP7
VEGFA
CNTF
STC1
BMP4
BMP2
GDF1
FLT3LG
TGFB2
CXCL12

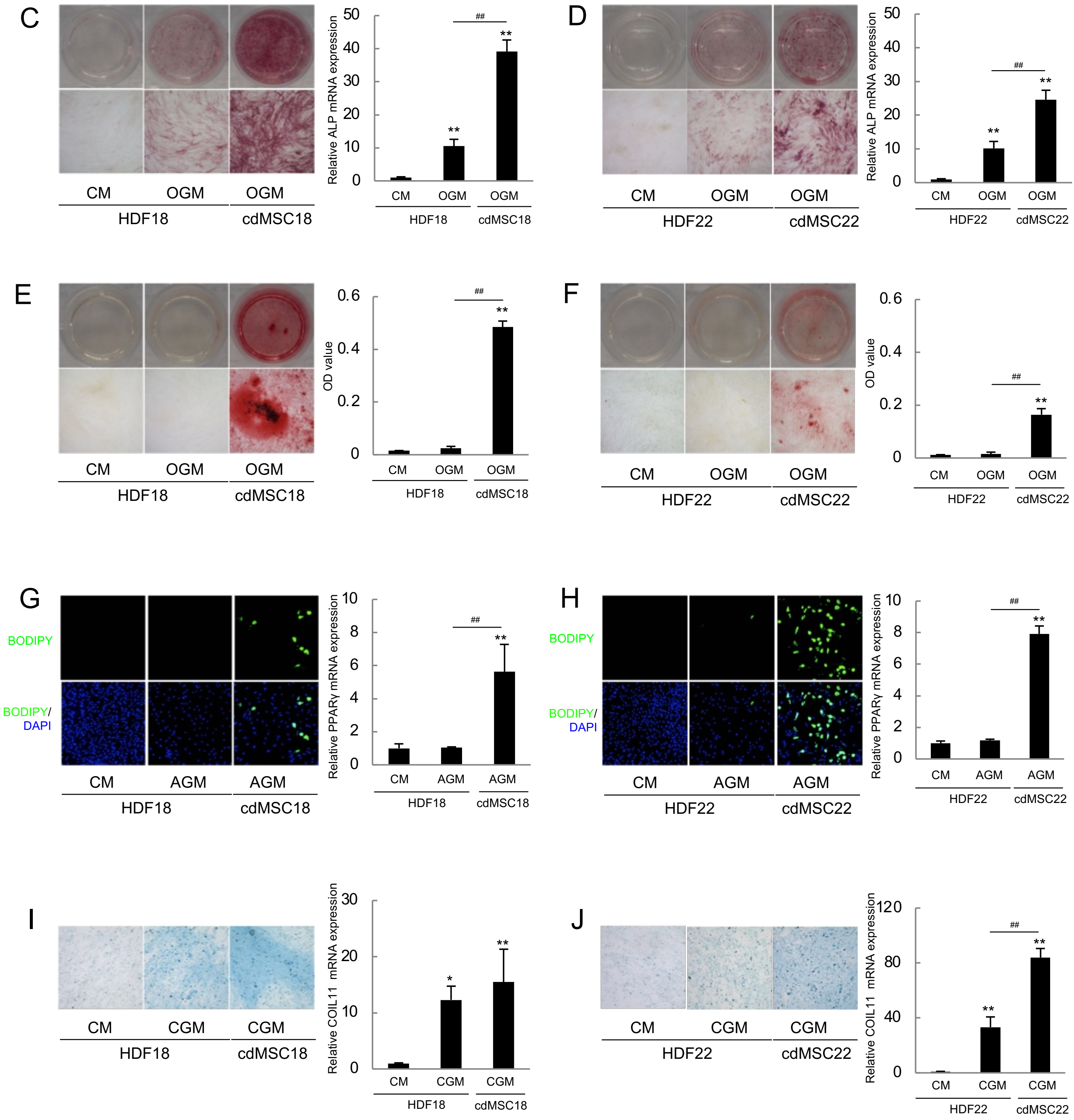

C
CM
OGM
OGM
HDF18
cdMSC18
Relative ALP mRNA expression
50
40
30
20
10
0
##
**
**
CM
OGM
OGM
HDF18
cdMSC18
D
CM
OGM
OGM
HDF22
cdMSC22
Relative ALP mRNA expression
50
40
30
20
10
0
##
**
**
CM
OGM
OGM
HDF22
cdMSC22
E
CM
OGM
OGM
HDF18
cdMSC18
OD value
0.6
0.4
0.2
0
##
**
CM
OGM
OGM
HDF18
cdMSC18
F
CM
OGM
OGM
HDF22
cdMSC22
OD value
0.6
0.4
0.2
0
##
**
CM
OGM
OGM
HDF22
cdMSC22
G
BODIPY
BODIPY/
DAPI
CM
AGM
AGM
HDF18
cdMSC18
Relative PPARγ mRNA expression
10
8
6
4
2
0
##
**
CM
AGM
AGM
HDF18
cdMSC18
H
BODIPY
BODIPY/
DAPI
CM
AGM
AGM
HDF22
cdMSC22
Relative PPARγ mRNA expression
10
8
6
4
2
0
##
**
CM
AGM
AGM
HDF22
cdMSC22
I
CM
CGM
CGM
HDF18
cdMSC18
Relative COIL11 mRNA expression
30
20
10
0
*
**
CM
CGM
CGM
HDF18
cdMSC18
J
CM
CGM
CGM
HDF22
cdMSC22
Relative COIL11 mRNA expression
120
80
40
0
##
**
**
CM
CGM
CGM
HDF22
cdMSC22

K

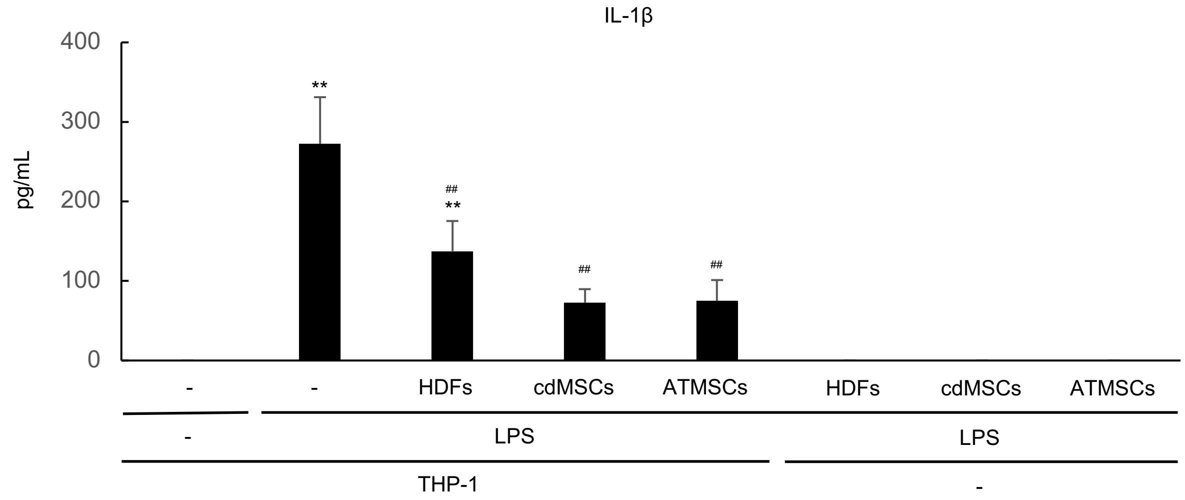
IL-1β
400
300
200
100
0
pg/mL
**
##
**
##
##
-
-
HDFs
cdMSCs
ATMSCs
HDFs
cdMSCs
ATMSCs
-
LPS
LPS
THP-1
-

L

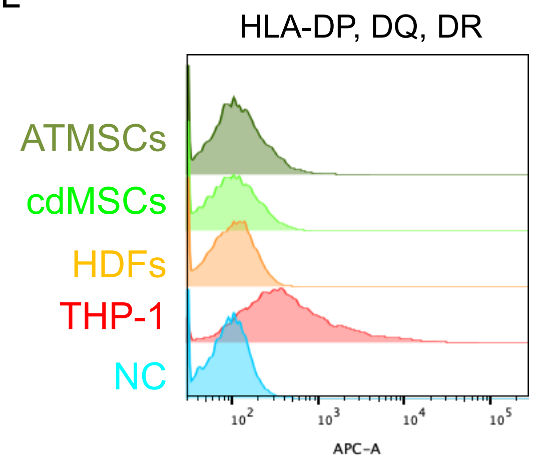
HLA-DP, DQ, DR
ATMSCs
cdMSCs
HDFs
THP-1
NC
10^2
10^3
10^4
10^5
APC-A

Fig. S3

A TGF-βR signal pathway

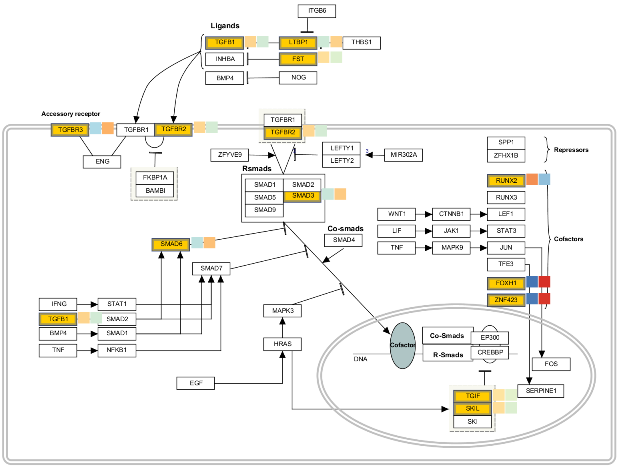

B WNT signal pathway

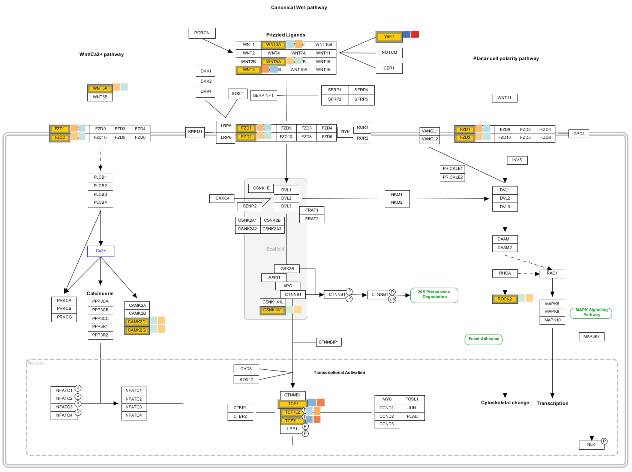

C MAPK pathway

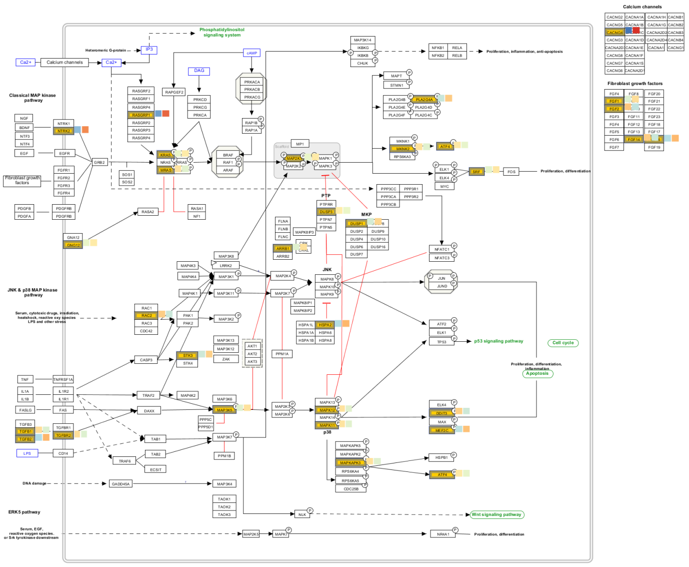

D Hedgehog pathway

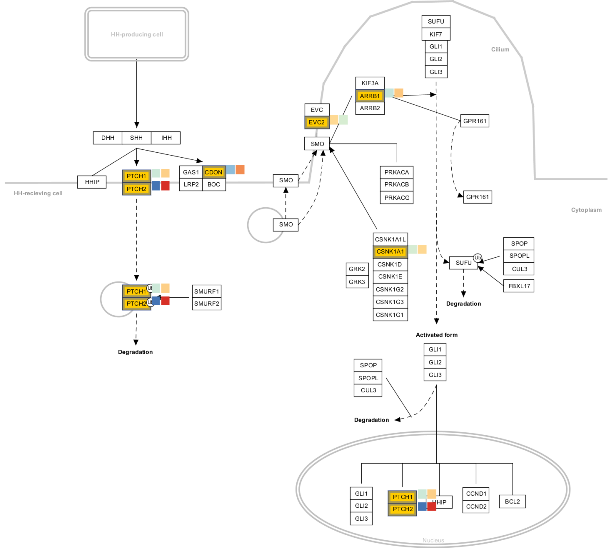

Fig. S4

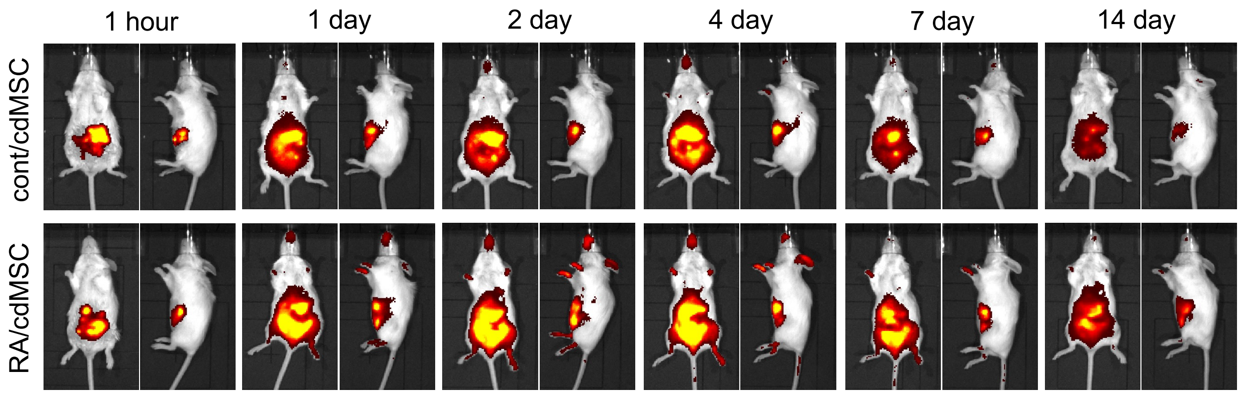

Table. S1

Antibody	Company	catalog number
FITC-anti Human CD31 anti-body	Becton Dickinson	555445
FITC-anti Human CD45 anti-bpdy	Becton Dickinson	555482
PE-anti Human CD34 anti-body	Becton Dickinson	550761
PE-anti Human CD146 anti-body	Becton Dickinson	550315
Pacific blue-anti Human CD73 anti-body	Biolegend	344011
APC-anti Human CD90 anti-body	Biolegend	328113
APC-anti Human CD105 anti-body	Biolegend	323207
APC-anti Human CD140b anti-body	Biolegend	323608
APC-anti Human CD271 anti-body	Becton Dickinson	560326
APC-anti Human HLA-DP, DQ, DR anti-body	Biolegend	361713
Anti-Mouse CD16/32 anti-body	Biolegend	101301
Pacific blue-anti Mouse CD3 anti-body	Biolegend	100213
APC-anti Mouse CD4 anti-body	Biolegend	100515
PE-anti Mouse IL-17A anti-body	Biolegend	506903
PE-anti Mouse FOXP3 anti-body	Biolegend	126403

Table. S2

Genes	Company	Sequence or Catalog Number
Human GAPDH	Applied Bioscience	F: 5’-CTCAAGATCATVAGCAATGCCTC-3'
		R: 5’-CCCACAGCCTTGGCAGC-3’
Human ALP	Applied Bioscience	F: 5’-TGACACCTGGAAGAGCTTCAAA-3’
		R: 5’-CCGTGCGGTTCCAGATG-3’
Human Runx2	Applied Bioscience	TaqMan™ Gene Expression Assay: Hs01047973_m1
Human Osteocalcin	Applied Bioscience	TaqMan™ Gene Expression Assay: Hs01587814_g1
Human ADIPOQ	Applied Bioscience	TaqMan™ Gene Expression Assay: Hs00605917_m1
Human PPARγ	Applied Bioscience	TaqMan™ Gene Expression Assay: Hs01115513_m1
Human COL2A1	Applied Bioscience	TaqMan™ Gene Expression Assay: Hs01060345_m1
Human COL11A1	Applied Bioscience	TaqMan™ Gene Expression Assay: Hs01097634_g1
Human CD34 up-stream	Applied Bioscience	F: 5’-TAGTGTCTTCCACTCGGTGCG-3'
		R: 5’-CGCGGGCGGTACTCAC-3’
Human CD140b up-stream	Applied Bioscience	F: 5’-GGAAACTGAGGCACTGAGAAATG-3'
		R: 5’-CCTCCCATCGCCCGTT-3’
Mouse β-actin	Applied Bioscience	TaqMan™ Gene Expression Assay: Mm02619580_g1
Mouse IL-1β	Applied Bioscience	TaqMan™ Gene Expression Assay: Mm01336189_m1
Mouse IL-6	Applied Bioscience	TaqMan™ Gene Expression Assay: Mm00446190_m1
Mouse IL-17A	Applied Bioscience	TaqMan™ Gene Expression Assay: Mm00439618_m1
Mouse TNF-α	Applied Bioscience	TaqMan™ Gene Expression Assay: Mm00443258_m1
Mouse TGF-β	Applied Bioscience	TaqMan™ Gene Expression Assay: Mm00436955_m1
Mouse Nox1	Applied Bioscience	TaqMan™ Gene Expression Assay: Mm00549170_m1
Mouse MPO	Applied Bioscience	TaqMan™ Gene Expression Assay: Mm01298424_m1
